# Aqua­(ethanol-κ*O*)tris­[4,4,4-tri­fluoro-1-(4-propoxyphen­yl)butane-1,3-dionato(1−)-κ^2^*O*,*O*′]europium(III)

**DOI:** 10.1107/S2414314626001380

**Published:** 2026-02-13

**Authors:** Tetsuji Moriguchi, Misa Sasaki, Noriko Miyoshi

**Affiliations:** a Department of Material Science, Faculty of Engineering, Kyushu Institute of, Technology, 1-1 Sensui-cho, Tobata-ku Kitakyushu, Fukuoka, Japan; b Technical Support Department, Management Headquarters, Kyushu Institute of, Technology, 1-1 Sensui-cho, Tobata-ku, Kitakyushu 804-8550, Japan; Vienna University of Technology, Austria

**Keywords:** crystal structure, europium(III) complex, co-ligand, ethanol ligand, hydrogen-bonding, fluorescence

## Abstract

In the title compound, [Eu(C_13_H_12_F_3_O_3_)_3_(C_2_H_5_OH)(H_2_O)], the tris­[4,4,4-tri­fluoro-1-(4-propoxyphen­yl)butane-1,3-dionato(1-)]europium(III) complex is augmented by ethanol and water mol­ecules as neutral co-ligands, leading to a coordination number of 8 of the central Eu^III^ ion. In the crystal structure, O—H⋯O hydrogen bonding involving ethanol and water as donor and meth­oxy O atoms as acceptor groups consolidates the crystal packing. The complex has been characterized by positive ion FAB-MS and luminescence spectroscopy.

## Abstract 2

The coordination number of the central Eu^III^ ion is 8, defined by three bidentate *β*-diketonato ligands, an aqua ligand and an ethanol ligand.

## Graphical abstract

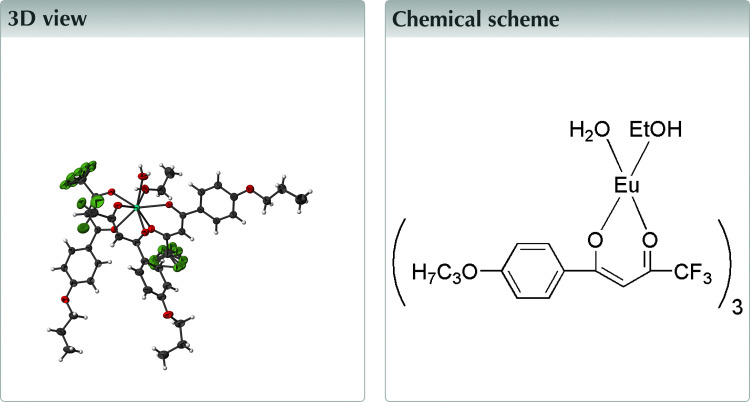

## Structure description

Tetra­kis(*β*-diketonato) lanthanide complexes can be synthesized by using an excess of the ligand (Moriguchi *et al.*, 2017*a* ▸,*b* ▸), while the title tris­(*β*-diketonato) europium(III) complex was obtained by adjusting the amount of the ligand during synthesis.

**Figure 1:**
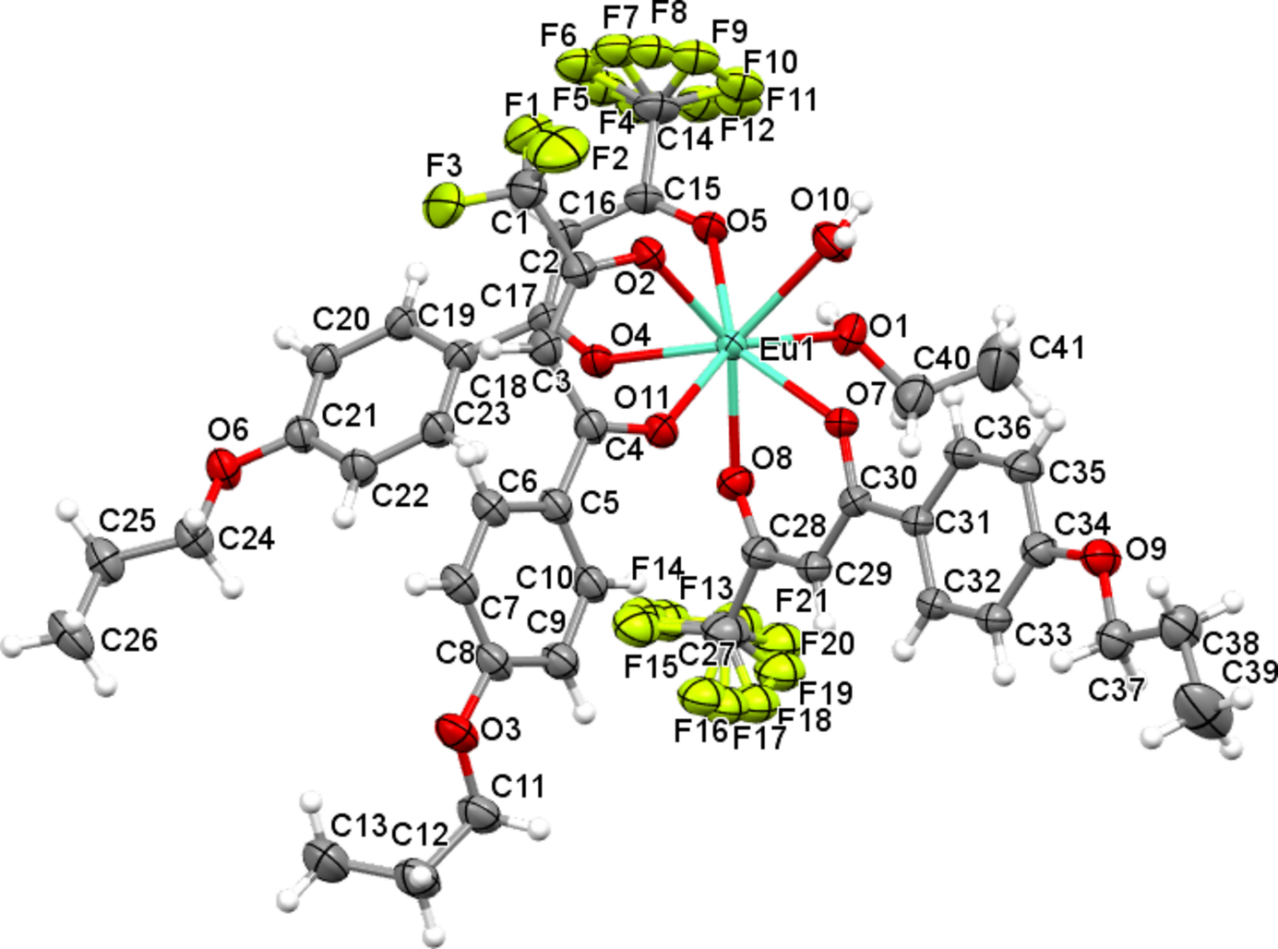
The mol­ecular structure of the title complex with displacement ellipsoids drawn at the 50% probability level; H atoms are shown as spheres of arbitrary radius.

**Figure 2:**
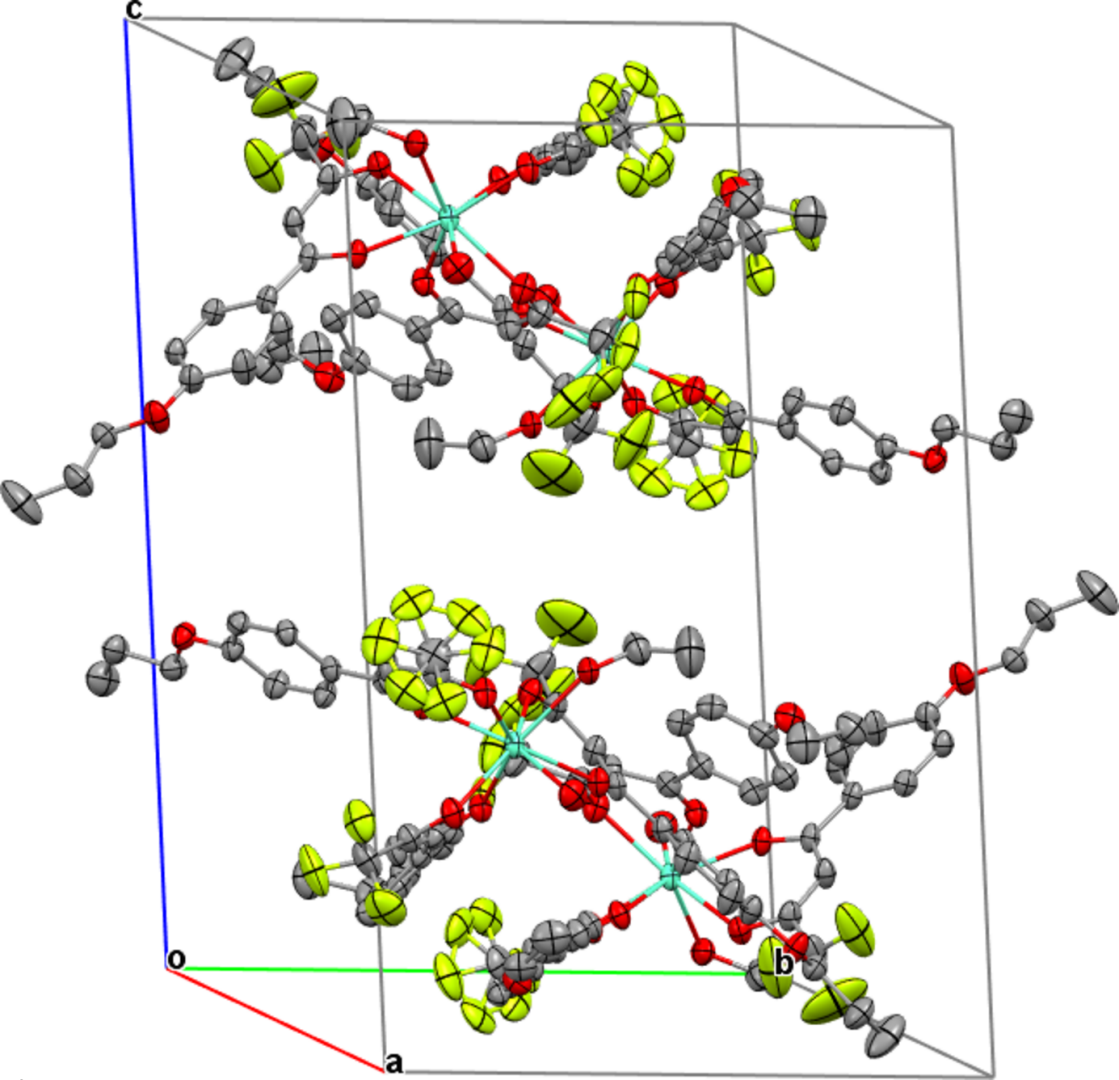
Crystal packing diagram of the title complex. Displacement ellipsoids are as in Fig. 1 ▸.

The title complex contains three (4′-propoxyphen­yl)-4,4,4-tri­fluoro-1,3-butane­dionate anions acting as bidentate ligands, and one water and one ethanol mol­ecule as neutral co-ligands, leading to a distorted eightfold coordination of the central Eu^III^ ion (Fig. 1 ▸). The Eu—O bond lengths involving the organic ligands are in a narrow range [2.340 (4)–2.385 (4) Å], while the Eu—O distances to the water and ethanol O atoms are considerably longer [2.488 (4) and 2.510 (4) Å]. The mol­ecular packing is shown in Fig. 2 ▸.

**Figure 3:**
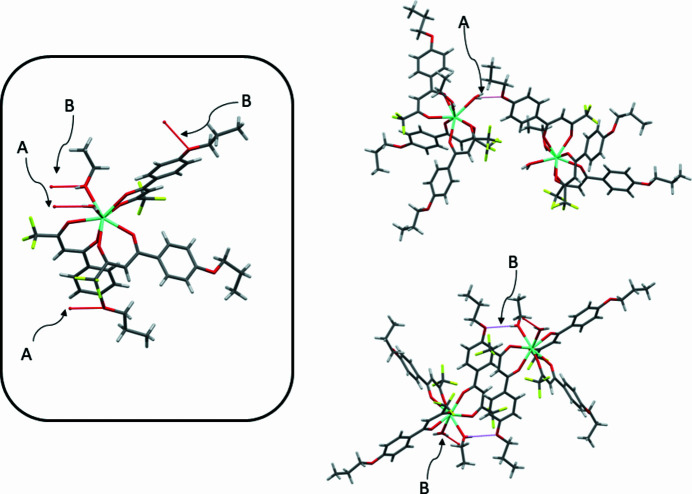
Capped stick representation of the title complex showing O—H⋯O hydrogen-bonding inter­actions as dashed lines. (A) Inter­molecular hydrogen-bonding network between aqua ligands and oxygen atoms of meth­oxy groups; (B) inter­molecular hydrogen bonding between H atoms of the ethanol ligands and oxygen atoms of meth­oxy groups.

In general, the coordination ability of an ethanol mol­ecule is rather weak, but the ethanol ligand of this complex is stabilized by intra- and inter­molecular hydrogen bonding between the hy­droxy group (O1—H1) and one of the *β*-diketonato O atoms (O5) of the same and one of the meth­oxy O atoms (O6) of an adjacent mol­ecule. Inter­estingly, only one H atom of the water mol­ecule forms hydrogen bonds (O10—H10*B*), namely to a meth­oxy-O atom (O9) of a neighboring mol­ecule (Table 1 ▸, Fig. 3 ▸).

**Figure 4:**
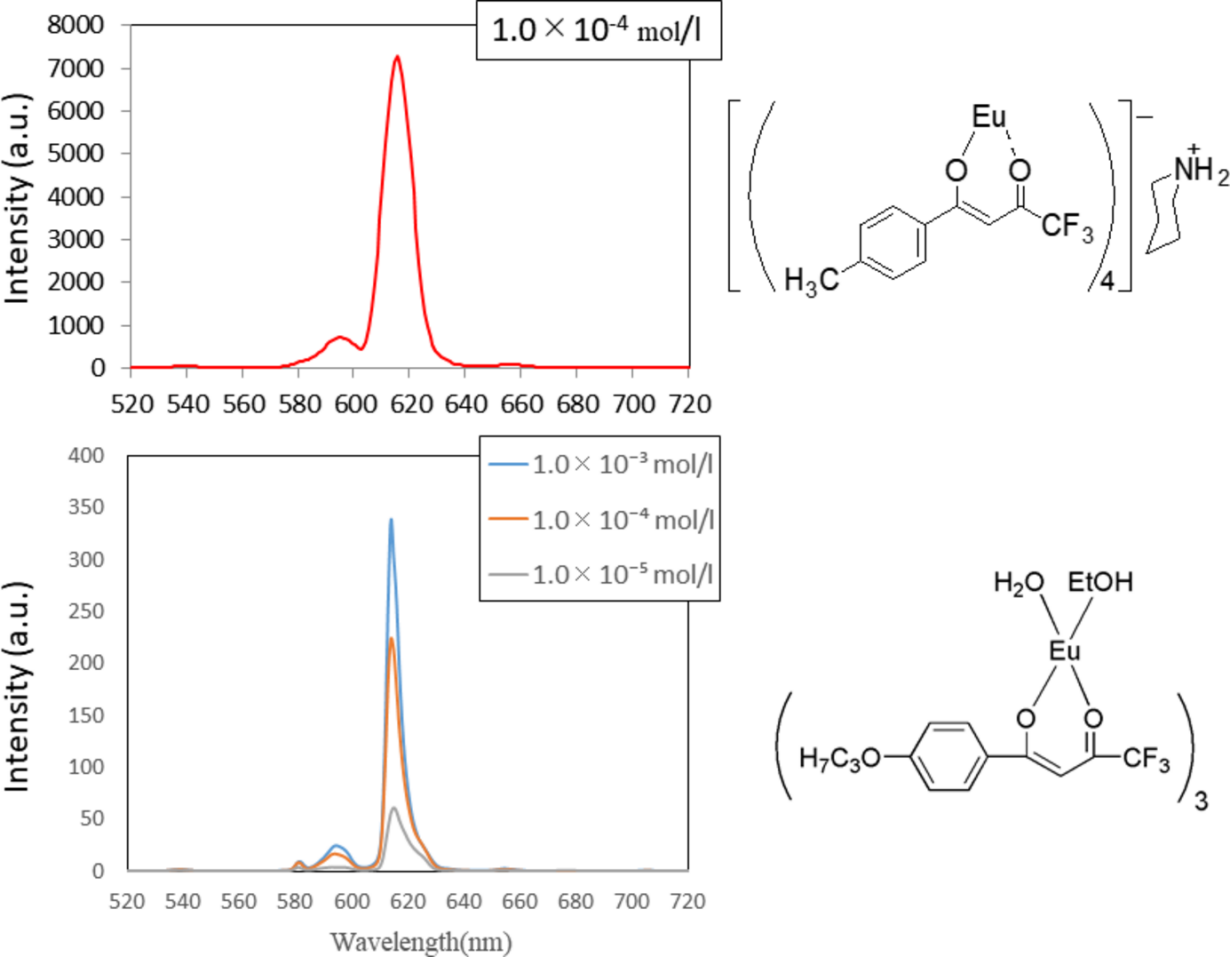
Fluorescence emission spectrum of the title complex (bottom) in comparison with a europium(III) complex having four *β*-diketonato ligands (top).

The fluorescence intensity from the trivalent europium ion is quite weak (Fig. 4 ▸) compared with a europium(III) complex having four *β*-diketonato ligands (Moriguchi *et al.*, 2017*a* ▸,*b* ▸). According to the previous investigations, the emission intensities from europium(III) is strong when the ligands have no hy­droxy group(*s*). This is due to the fact that the excitation energy level (^5^*D*_0_) of the 4*f* orbital of the central europium(III) ion is effectively quenched by O—H vibrations of the ethanol and water ligands.

## Synthesis and crystallization

The title complex was synthesized in two steps. In the first step, the *β*-diketone ligand (4′-propoxyphen­yl)-4,4,4-tri­fluoro-1,3-butane­dione was prepared by Claisen condensation of 4′-prop­oxy aceto­phenone with ethyl tri­fluoro­acetate using sodium hydride as a base catalyst in tetra­hydro­furan as solvent. In the second step, the obtained ligand was reacted with europium(III) chloride in the presence of pyridine as a base in ethanol as a solvent in analogy to the method reported in the literature (Moriguchi *et al.*, 2017*a* ▸,*b* ▸), yielding the corresponding complex in good yield (85%). The complex is stable under air and moisture conditions. Colorless needles of the title complex were obtained by slow evaporation of a toluene solution of the crude reaction product at room temperature. Analysis: positive ion FAB-MS: 1035(*M*H^+^), 1017(*M*H^+^—H_2_O), 973(*M*H^+^—H_2_O—EtOH).

A HITACHI F-2500 spectrophotometer (Hitachi High-Technologies Corporation, Tokyo, Japan) was used for fluorescence spectra measurements in the 250–900 nm range. The fluorescence measurements were carried out in di­chloro­methane solution (1 × 10^−3^–10^−5^ mol l^−1^).

## Refinement

Crystal data, data collection and structure refinement details are summarized in Table 2 ▸. The F atoms of two of the three CF_3_ groups are positionally disordered over three sets of sites and were refined with similarity restraints on bond lengths and displacement parameters and a fixed occupancy of 1/3 for each F atom. The high remaining electron density near O1 (0.83 Å) of the ethanol ligand suggests that this mol­ecule is potentially disordered over two sets of sites. However, based on the present X-ray diffraction data, disorder could not be modelled convincingly and thus was not considered for the final model.

## Supplementary Material

Crystal structure: contains datablock(s) I. DOI: 10.1107/S2414314626001380/wm4241sup1.cif

Structure factors: contains datablock(s) I. DOI: 10.1107/S2414314626001380/wm4241Isup2.hkl

CCDC reference: 2530119

Additional supporting information:  crystallographic information; 3D view; checkCIF report

## Figures and Tables

**Table 1:** Hydrogen-bond geometry (Å, °)

*D*—H⋯*A*	*D*—H	H⋯*A*	*D*⋯*A*	*D*—H⋯*A*
O1—H1⋯O5	0.84	2.47	2.761 (5)	101
O1—H1⋯O6^i^	0.84	2.17	2.978 (6)	161
O10—H10*B*⋯O9^ii^	0.85	2.15	2.866 (6)	142

Symmetry codes: (i) 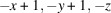; (ii) 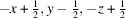.

**Table 2:** Experimental details

Crystal data	
Chemical formula	[Eu(C_13_H_12_F_3_O_3_)_3_(C_2_H_6_O)(H_2_O)]
*M*_r_	1035.72
Crystal system, space group	Monoclinic, *P*2_1_/*n*
Temperature (K)	90
*a*, *b*, *c* (Å)	13.895 (11), 15.268 (12), 22.326 (17)
β (°)	107.872 (7)
*V* (Å^3^)	4508 (6)
*Z*	4
Radiation type	Mo *K*α
μ (mm^−1^)	1.48
Crystal size (mm)	0.35 × 0.25 × 0.25

Data collection	
Diffractometer	Bruker APEXII CCD
Absorption correction	Multi-scan (*SADABS*; Krause *et al.*, 2015 ▸)
*T*_min_, *T*_max_	0.373, 0.746
No. of measured, independent and observed [*I* > 2σ(*I*)] reflections	46416, 10679, 8033
*R*_int_	0.066
(sin θ/λ)_max_ (Å^−1^)	0.669

Refinement	
*R*[*F*^2^ > 2σ(*F*^2^)], *wR*(*F*^2^), *S*	0.052, 0.138, 1.07
No. of reflections	10679
No. of parameters	673
No. of restraints	699
H-atom treatment	H-atom parameters constrained
Δρ_max_, Δρ_min_ (e Å^−3^)	2.15, −1.78

Computer programs: *APEX2* and *SAINT* (Bruker, 2009 ▸), *OLEX2.solve* (Bourhis *et al.*, 2015 ▸), *SHELXL* (Sheldrick, 2015 ▸), *OLEX2* (Dolomanov *et al.*, 2009 ▸), *Mercury* (Macrae *et al.*, 2020 ▸) and *publCIF* (Westrip, 2010 ▸).

## full crystallographic data

### Aqua(ethanol-κO)tris[4,4,4-trifluoro-1-(4-propoxyphenyl)butane-1,3-dionato(1-)-κ2O,O']europium(III) Crystal data

**Table:**

[Eu(C_13_H_12_F_3_O_3_)_3_(C_2_H_6_O)(H_2_O)]	*F*(000) = 2088
*M**_r_* = 1035.72	*D*_x_ = 1.526 Mg m^−^^3^
Monoclinic, *P*2_1_/*n*	Mo *K*α radiation, λ = 0.71073 Å
*a* = 13.895 (11) Å	Cell parameters from 9870 reflections
*b* = 15.268 (12) Å	θ = 2.4–28.4°
*c* = 22.326 (17) Å	µ = 1.48 mm^−^^1^
β = 107.872 (7)°	*T* = 90 K
*V* = 4508 (6) Å^3^	Prism, clear light yellow
*Z* = 4	0.35 × 0.25 × 0.25 mm

### Aqua(ethanol-κO)tris[4,4,4-trifluoro-1-(4-propoxyphenyl)butane-1,3-dionato(1-)-κ2O,O']europium(III) Data collection

**Table:**

Bruker APEXII CCD diffractometer	8033 reflections with *I* > 2σ(*I*)
Graphite monochromator	*R*_int_ = 0.066
φ and ω scans	θ_max_ = 28.4°, θ_min_ = 1.9°
Absorption correction: multi-scan (SADABS; Krause *et al.*, 2015)	*h* = −18→18
*T*_min_ = 0.373, *T*_max_ = 0.746	*k* = −19→20
46416 measured reflections	*l* = −29→29
10679 independent reflections	

### Aqua(ethanol-κO)tris[4,4,4-trifluoro-1-(4-propoxyphenyl)butane-1,3-dionato(1-)-κ2O,O']europium(III) Refinement

**Table:**

Refinement on *F*^2^	Primary atom site location: iterative
Least-squares matrix: full	Hydrogen site location: mixed
*R*[*F*^2^ > 2σ(*F*^2^)] = 0.052	H-atom parameters constrained
*wR*(*F*^2^) = 0.138	*w* = 1/[σ^2^(*F*_o_^2^) + (0.0417*P*)^2^ + 21.805*P*] where *P* = (*F*_o_^2^ + 2*F*_c_^2^)/3
*S* = 1.07	(Δ/σ)_max_ = 0.001
10679 reflections	Δρ_max_ = 2.15 e Å^−^^3^
673 parameters	Δρ_min_ = −1.78 e Å^−^^3^
699 restraints	

### Aqua(ethanol-κO)tris[4,4,4-trifluoro-1-(4-propoxyphenyl)butane-1,3-dionato(1-)-κ2O,O']europium(III) Special details

**Table:**

Geometry. All esds (except the esd in the dihedral angle between two l.s. planes) are estimated using the full covariance matrix. The cell esds are taken into account individually in the estimation of esds in distances, angles and torsion angles; correlations between esds in cell parameters are only used when they are defined by crystal symmetry. An approximate (isotropic) treatment of cell esds is used for estimating esds involving l.s. planes.

### Aqua(ethanol-κO)tris[4,4,4-trifluoro-1-(4-propoxyphenyl)butane-1,3-dionato(1-)-κ2O,O']europium(III) Fractional atomic coordinates and isotropic or equivalent isotropic displacement parameters (Å^2^)

**Table:**

	*x*	*y*	*z*	*U*_iso_*/*U*_eq_	Occ. (<1)
Eu1	0.38949 (2)	0.69722 (2)	0.14284 (2)	0.02935 (9)	
F1	0.3292 (3)	0.4156 (3)	0.2184 (2)	0.0717 (12)	
F2	0.3598 (4)	0.4628 (3)	0.3121 (2)	0.0843 (14)	
F3	0.4716 (3)	0.3871 (3)	0.2858 (3)	0.0861 (15)	
F4	0.1483 (8)	0.4658 (9)	−0.0363 (6)	0.0515 (13)	0.3333
F5	0.1605 (8)	0.4260 (8)	−0.0152 (6)	0.0523 (14)	0.3333
F6	0.1651 (8)	0.3989 (8)	0.0223 (7)	0.0532 (13)	0.3333
F7	0.1404 (8)	0.4283 (8)	0.0557 (6)	0.0525 (14)	0.3333
F8	0.1272 (9)	0.4621 (9)	0.0731 (6)	0.0507 (14)	0.3333
F9	0.1038 (8)	0.5029 (9)	0.0632 (6)	0.0537 (13)	0.3333
F10	0.0873 (9)	0.5513 (9)	0.0169 (6)	0.0534 (14)	0.3333
F11	0.0961 (9)	0.5553 (10)	−0.0082 (6)	0.0502 (14)	0.3333
F12	0.1152 (9)	0.5192 (9)	−0.0255 (6)	0.0546 (14)	0.3333
F13	0.6668 (11)	0.7711 (9)	0.0579 (6)	0.0582 (13)	0.3333
F14	0.6854 (12)	0.7532 (12)	0.0810 (6)	0.0560 (15)	0.3333
F15	0.7047 (11)	0.7542 (11)	0.1024 (6)	0.0575 (14)	0.3333
F16	0.7472 (13)	0.8550 (10)	0.1392 (8)	0.0576 (15)	0.3333
F17	0.7397 (14)	0.8772 (10)	0.1287 (8)	0.0567 (14)	0.3333
F18	0.7114 (10)	0.8999 (9)	0.1038 (7)	0.0582 (14)	0.3333
F19	0.6482 (10)	0.8988 (9)	0.0552 (7)	0.0599 (13)	0.3333
F20	0.6140 (10)	0.8807 (10)	0.0329 (7)	0.0597 (14)	0.3333
F21	0.6177 (10)	0.8324 (10)	0.0282 (6)	0.0603 (14)	0.3333
O1	0.2789 (3)	0.7845 (2)	0.05251 (18)	0.0417 (8)	
H1	0.260997	0.750020	0.021750	0.063*	
O2	0.3749 (3)	0.5851 (2)	0.21394 (18)	0.0402 (8)	
O3	1.0092 (3)	0.6872 (3)	0.3771 (2)	0.0475 (9)	
O4	0.4726 (2)	0.5815 (2)	0.11072 (17)	0.0346 (7)	
O5	0.2649 (2)	0.6080 (2)	0.07535 (18)	0.0390 (8)	
O6	0.7832 (3)	0.2999 (2)	0.07384 (18)	0.0397 (8)	
O7	0.4154 (2)	0.8416 (2)	0.18384 (17)	0.0338 (7)	
O8	0.5064 (3)	0.7671 (2)	0.09959 (18)	0.0390 (8)	
O9	0.4575 (3)	1.1677 (3)	0.3617 (2)	0.0463 (8)	
O10	0.2435 (3)	0.7377 (3)	0.1797 (2)	0.0494 (10)	
H10A	0.244983	0.752326	0.217225	0.074*	
H10B	0.181341	0.740218	0.158069	0.074*	
O11	0.5433 (3)	0.6904 (2)	0.22645 (17)	0.0363 (8)	
C1	0.4009 (5)	0.4499 (4)	0.2665 (3)	0.0515 (13)	
C2	0.4427 (4)	0.5358 (4)	0.2479 (3)	0.0398 (10)	
C3	0.5461 (4)	0.5499 (4)	0.2719 (3)	0.0395 (10)	
H3	0.587672	0.504971	0.295879	0.047*	
C4	0.5923 (4)	0.6301 (3)	0.2615 (2)	0.0349 (9)	
C5	0.7017 (4)	0.6454 (4)	0.2933 (2)	0.0355 (9)	
C6	0.7591 (4)	0.5950 (4)	0.3444 (2)	0.0372 (10)	
H6	0.727560	0.548968	0.360085	0.045*	
C7	0.8611 (4)	0.6115 (4)	0.3721 (3)	0.0398 (10)	
H7	0.899074	0.577492	0.406944	0.048*	
C8	0.9084 (4)	0.6787 (4)	0.3485 (3)	0.0405 (10)	
C9	0.8517 (4)	0.7310 (4)	0.2986 (3)	0.0405 (10)	
H9	0.882673	0.777805	0.283291	0.049*	
C10	0.7499 (4)	0.7134 (4)	0.2721 (3)	0.0390 (10)	
H10	0.711314	0.748848	0.238289	0.047*	
C11	1.0656 (4)	0.7462 (4)	0.3500 (3)	0.0497 (12)	
H11A	1.047797	0.807734	0.355700	0.060*	
H11B	1.050643	0.734523	0.304458	0.060*	
C12	1.1768 (4)	0.7293 (5)	0.3846 (3)	0.0543 (14)	
H12A	1.218635	0.772369	0.370517	0.065*	
H12B	1.189338	0.737475	0.430309	0.065*	
C13	1.2080 (5)	0.6371 (5)	0.3724 (4)	0.070 (2)	
H13A	1.200410	0.630323	0.327540	0.105*	
H13B	1.164856	0.594437	0.384721	0.105*	
H13C	1.278703	0.627240	0.397111	0.105*	
C14	0.1625 (4)	0.4900 (4)	0.0259 (3)	0.0492 (9)	
C15	0.2677 (4)	0.5306 (3)	0.0550 (3)	0.0376 (9)	
C16	0.3496 (4)	0.4796 (3)	0.0564 (3)	0.0365 (10)	
H16	0.338196	0.422403	0.038959	0.044*	
C17	0.4515 (4)	0.5091 (3)	0.0831 (2)	0.0331 (9)	
C18	0.5375 (4)	0.4528 (3)	0.0808 (2)	0.0324 (9)	
C19	0.5262 (4)	0.3785 (3)	0.0421 (2)	0.0340 (9)	
H19	0.460527	0.362330	0.016236	0.041*	
C20	0.6083 (4)	0.3284 (3)	0.0408 (2)	0.0344 (9)	
H20	0.598952	0.278207	0.014443	0.041*	
C21	0.7055 (4)	0.3519 (3)	0.0786 (2)	0.0351 (9)	
C22	0.7185 (4)	0.4258 (4)	0.1171 (3)	0.0390 (10)	
H22	0.784110	0.442024	0.142889	0.047*	
C23	0.6349 (4)	0.4754 (3)	0.1176 (2)	0.0369 (10)	
H23	0.644292	0.525898	0.143617	0.044*	
C24	0.8825 (4)	0.3155 (4)	0.1173 (3)	0.0407 (10)	
H24A	0.880112	0.312504	0.161162	0.049*	
H24B	0.906614	0.374409	0.110229	0.049*	
C25	0.9531 (4)	0.2463 (4)	0.1068 (3)	0.0459 (12)	
H25A	0.952113	0.247728	0.062273	0.055*	
H25B	0.929492	0.187784	0.115306	0.055*	
C26	1.0606 (5)	0.2610 (6)	0.1494 (3)	0.0629 (18)	
H26A	1.085889	0.316960	0.138773	0.094*	
H26B	1.104056	0.213346	0.143466	0.094*	
H26C	1.061166	0.262188	0.193386	0.094*	
C27	0.6537 (5)	0.8347 (5)	0.0915 (3)	0.0558 (10)	
C28	0.5678 (4)	0.8289 (4)	0.1220 (3)	0.0393 (9)	
C29	0.5662 (4)	0.8905 (3)	0.1669 (3)	0.0366 (9)	
H29	0.620230	0.931344	0.179335	0.044*	
C30	0.4877 (4)	0.8962 (3)	0.1958 (2)	0.0327 (9)	
C31	0.4862 (4)	0.9686 (3)	0.2402 (2)	0.0330 (9)	
C32	0.5648 (4)	1.0294 (3)	0.2623 (2)	0.0329 (9)	
H32	0.623274	1.025166	0.248902	0.040*	
C33	0.5583 (4)	1.0961 (3)	0.3036 (2)	0.0345 (9)	
H33	0.612308	1.136543	0.318478	0.041*	
C34	0.4720 (4)	1.1033 (4)	0.3230 (3)	0.0407 (9)	
C35	0.3930 (4)	1.0432 (4)	0.3016 (3)	0.0452 (11)	
H35	0.334273	1.048029	0.314656	0.054*	
C36	0.4011 (4)	0.9765 (4)	0.2613 (3)	0.0410 (10)	
H36	0.347744	0.935121	0.247508	0.049*	
C37	0.5385 (4)	1.2269 (4)	0.3904 (3)	0.0432 (11)	
H37A	0.554866	1.262618	0.357822	0.052*	
H37B	0.599709	1.193830	0.413954	0.052*	
C38	0.5041 (5)	1.2847 (4)	0.4343 (3)	0.0528 (13)	
H38A	0.489114	1.247955	0.466894	0.063*	
H38B	0.440773	1.314647	0.410382	0.063*	
C39	0.5820 (6)	1.3529 (6)	0.4662 (4)	0.091 (3)	
H39A	0.646328	1.323980	0.487691	0.136*	
H39B	0.558601	1.385239	0.497069	0.136*	
H39C	0.591566	1.393675	0.434636	0.136*	
C40	0.2950 (5)	0.8715 (4)	0.0305 (3)	0.0528 (13)	
H40A	0.355519	0.898110	0.060798	0.063*	
H40B	0.307818	0.866406	−0.010562	0.063*	
C41	0.2088 (7)	0.9280 (5)	0.0238 (5)	0.097 (3)	
H41A	0.193969	0.931113	0.063900	0.146*	
H41B	0.150012	0.904426	−0.008739	0.146*	
H41C	0.224040	0.986847	0.011608	0.146*	

### Aqua(ethanol-κO)tris[4,4,4-trifluoro-1-(4-propoxyphenyl)butane-1,3-dionato(1-)-κ2O,O']europium(III) Atomic displacement parameters (Å^2^)

**Table:**

	*U*^11^	*U*^22^	*U*^33^	*U*^12^	*U*^13^	*U*^23^
Eu1	0.02182 (12)	0.02491 (13)	0.03887 (15)	0.00024 (9)	0.00569 (9)	−0.00040 (10)
F1	0.059 (2)	0.045 (2)	0.101 (3)	−0.0149 (18)	0.011 (2)	0.009 (2)
F2	0.088 (3)	0.091 (3)	0.088 (3)	−0.015 (3)	0.047 (3)	0.024 (3)
F3	0.052 (2)	0.050 (2)	0.145 (4)	0.0066 (18)	0.012 (2)	0.048 (3)
F4	0.034 (2)	0.051 (3)	0.060 (3)	−0.014 (2)	0.002 (2)	−0.006 (2)
F5	0.036 (2)	0.051 (3)	0.062 (3)	−0.015 (2)	0.003 (2)	−0.007 (2)
F6	0.036 (2)	0.050 (3)	0.065 (3)	−0.015 (2)	0.003 (2)	−0.005 (2)
F7	0.036 (2)	0.053 (3)	0.063 (3)	−0.017 (2)	0.007 (2)	−0.002 (2)
F8	0.034 (2)	0.050 (3)	0.064 (3)	−0.013 (2)	0.008 (2)	−0.001 (2)
F9	0.035 (2)	0.056 (3)	0.065 (3)	−0.014 (2)	0.008 (2)	−0.005 (2)
F10	0.032 (2)	0.054 (3)	0.066 (3)	−0.011 (2)	0.003 (2)	−0.004 (3)
F11	0.032 (2)	0.052 (3)	0.060 (3)	−0.011 (2)	0.004 (2)	−0.004 (2)
F12	0.036 (2)	0.054 (3)	0.063 (3)	−0.013 (2)	−0.001 (2)	−0.002 (2)
F13	0.055 (2)	0.061 (2)	0.069 (3)	−0.008 (2)	0.035 (2)	−0.011 (2)
F14	0.052 (2)	0.060 (3)	0.067 (3)	−0.005 (2)	0.034 (2)	−0.012 (3)
F15	0.052 (2)	0.061 (2)	0.070 (3)	−0.005 (2)	0.034 (2)	−0.011 (2)
F16	0.050 (2)	0.061 (3)	0.071 (3)	−0.011 (2)	0.032 (2)	−0.009 (2)
F17	0.050 (2)	0.060 (3)	0.070 (3)	−0.011 (2)	0.032 (2)	−0.008 (2)
F18	0.054 (2)	0.060 (2)	0.070 (3)	−0.013 (2)	0.033 (2)	−0.010 (2)
F19	0.055 (2)	0.063 (2)	0.070 (3)	−0.011 (2)	0.033 (2)	−0.007 (2)
F20	0.058 (2)	0.061 (3)	0.070 (3)	−0.013 (2)	0.033 (2)	−0.009 (2)
F21	0.058 (2)	0.064 (3)	0.069 (3)	−0.008 (2)	0.035 (2)	−0.009 (2)
O1	0.050 (2)	0.0285 (18)	0.045 (2)	0.0042 (15)	0.0119 (17)	0.0036 (15)
O2	0.0274 (16)	0.0380 (18)	0.054 (2)	0.0035 (14)	0.0102 (15)	0.0085 (16)
O3	0.0271 (16)	0.059 (2)	0.0521 (19)	−0.0005 (15)	0.0056 (14)	−0.0019 (17)
O4	0.0260 (15)	0.0299 (16)	0.0447 (18)	−0.0014 (13)	0.0064 (13)	−0.0077 (14)
O5	0.0240 (15)	0.0331 (18)	0.053 (2)	−0.0017 (13)	0.0008 (14)	−0.0026 (15)
O6	0.0330 (16)	0.0412 (17)	0.0415 (17)	0.0080 (14)	0.0065 (14)	−0.0062 (14)
O7	0.0257 (15)	0.0275 (16)	0.0493 (19)	−0.0021 (13)	0.0129 (14)	0.0001 (14)
O8	0.0373 (17)	0.0333 (17)	0.0490 (19)	−0.0050 (15)	0.0170 (15)	−0.0077 (15)
O9	0.0414 (17)	0.0415 (18)	0.063 (2)	−0.0043 (15)	0.0259 (16)	−0.0168 (16)
O10	0.034 (2)	0.056 (3)	0.060 (3)	0.0085 (19)	0.0189 (18)	0.004 (2)
O11	0.0280 (15)	0.0325 (17)	0.0431 (18)	0.0010 (13)	0.0031 (14)	0.0009 (14)
C1	0.041 (2)	0.046 (3)	0.064 (3)	0.001 (2)	0.010 (2)	0.018 (2)
C2	0.0325 (18)	0.037 (2)	0.050 (2)	0.0030 (16)	0.0124 (17)	0.0087 (18)
C3	0.032 (2)	0.035 (2)	0.047 (2)	0.0028 (17)	0.0050 (18)	0.0042 (19)
C4	0.0288 (17)	0.0340 (19)	0.0390 (19)	0.0040 (16)	0.0063 (16)	−0.0026 (16)
C5	0.0268 (17)	0.0382 (19)	0.0398 (19)	0.0043 (16)	0.0078 (16)	−0.0025 (17)
C6	0.0278 (19)	0.043 (2)	0.040 (2)	0.0036 (18)	0.0088 (17)	0.0004 (19)
C7	0.0292 (19)	0.049 (2)	0.039 (2)	0.0060 (18)	0.0070 (17)	0.0002 (19)
C8	0.0263 (17)	0.049 (2)	0.044 (2)	0.0045 (16)	0.0076 (16)	−0.0055 (18)
C9	0.031 (2)	0.044 (2)	0.045 (2)	0.0008 (18)	0.0093 (18)	−0.003 (2)
C10	0.0292 (19)	0.042 (2)	0.043 (2)	0.0052 (17)	0.0066 (17)	0.0003 (19)
C11	0.030 (2)	0.058 (3)	0.057 (3)	−0.002 (2)	0.007 (2)	−0.005 (2)
C12	0.033 (2)	0.063 (3)	0.061 (3)	−0.004 (2)	0.006 (2)	−0.008 (3)
C13	0.038 (3)	0.067 (5)	0.099 (6)	0.003 (3)	0.014 (3)	−0.002 (4)
C14	0.0319 (16)	0.049 (2)	0.060 (2)	−0.0134 (15)	0.0055 (15)	−0.0040 (18)
C15	0.0270 (17)	0.0342 (19)	0.048 (2)	−0.0074 (16)	0.0068 (16)	−0.0024 (17)
C16	0.0296 (19)	0.0266 (19)	0.050 (2)	−0.0043 (16)	0.0076 (17)	−0.0042 (18)
C17	0.0290 (17)	0.0269 (17)	0.0411 (19)	−0.0035 (15)	0.0075 (15)	−0.0016 (16)
C18	0.0296 (17)	0.0259 (17)	0.0396 (19)	−0.0011 (15)	0.0076 (16)	−0.0014 (16)
C19	0.0316 (19)	0.0277 (19)	0.039 (2)	−0.0009 (16)	0.0056 (17)	−0.0009 (17)
C20	0.035 (2)	0.0290 (19)	0.036 (2)	0.0004 (17)	0.0066 (17)	−0.0029 (17)
C21	0.0324 (18)	0.0330 (19)	0.0379 (19)	0.0038 (16)	0.0077 (16)	−0.0020 (16)
C22	0.032 (2)	0.037 (2)	0.044 (2)	0.0002 (18)	0.0062 (18)	−0.0076 (18)
C23	0.032 (2)	0.032 (2)	0.043 (2)	0.0007 (17)	0.0060 (17)	−0.0086 (18)
C24	0.033 (2)	0.048 (2)	0.041 (2)	0.0063 (19)	0.0112 (18)	−0.0014 (19)
C25	0.039 (2)	0.053 (3)	0.047 (3)	0.011 (2)	0.016 (2)	−0.001 (2)
C26	0.037 (3)	0.082 (5)	0.070 (4)	0.017 (3)	0.019 (3)	0.005 (4)
C27	0.0514 (18)	0.057 (2)	0.069 (2)	−0.0097 (16)	0.0330 (17)	−0.0111 (18)
C28	0.0347 (18)	0.0352 (19)	0.055 (2)	−0.0041 (16)	0.0237 (17)	−0.0051 (17)
C29	0.0316 (19)	0.030 (2)	0.053 (2)	−0.0039 (16)	0.0190 (17)	−0.0046 (18)
C30	0.0276 (17)	0.0245 (17)	0.0475 (19)	−0.0010 (15)	0.0138 (16)	0.0011 (16)
C31	0.0273 (17)	0.0267 (18)	0.048 (2)	−0.0006 (15)	0.0160 (16)	0.0017 (16)
C32	0.0264 (18)	0.031 (2)	0.044 (2)	−0.0011 (16)	0.0137 (16)	0.0006 (17)
C33	0.0299 (19)	0.032 (2)	0.045 (2)	−0.0040 (17)	0.0160 (17)	−0.0019 (18)
C34	0.0376 (19)	0.0354 (19)	0.055 (2)	−0.0028 (17)	0.0229 (17)	−0.0074 (18)
C35	0.037 (2)	0.039 (2)	0.068 (3)	−0.0065 (18)	0.0274 (19)	−0.011 (2)
C36	0.0311 (19)	0.035 (2)	0.062 (2)	−0.0062 (17)	0.0215 (18)	−0.007 (2)
C37	0.042 (2)	0.039 (2)	0.049 (2)	−0.0012 (19)	0.0161 (19)	−0.011 (2)
C38	0.052 (3)	0.050 (3)	0.056 (3)	−0.002 (2)	0.016 (2)	−0.019 (2)
C39	0.054 (4)	0.102 (6)	0.103 (6)	−0.004 (4)	0.005 (4)	−0.064 (5)
C40	0.058 (3)	0.040 (3)	0.057 (3)	0.002 (2)	0.012 (2)	0.006 (2)
C41	0.083 (6)	0.049 (4)	0.150 (8)	0.015 (4)	0.020 (6)	0.001 (5)

### Aqua(ethanol-κO)tris[4,4,4-trifluoro-1-(4-propoxyphenyl)butane-1,3-dionato(1-)-κ2O,O']europium(III) Geometric parameters (Å, º)

**Table:**

Eu1—O1	2.510 (4)	C3—H3	0.9500
Eu1—O2	2.385 (4)	C3—C4	1.433 (7)
Eu1—O4	2.340 (4)	C4—C5	1.486 (7)
Eu1—O5	2.349 (4)	C5—C6	1.403 (7)
Eu1—O7	2.371 (4)	C5—C10	1.394 (8)
Eu1—O8	2.382 (4)	C6—H6	0.9500
Eu1—O10	2.488 (4)	C6—C7	1.385 (7)
Eu1—O11	2.370 (4)	C7—H7	0.9500
F1—C1	1.327 (8)	C7—C8	1.403 (8)
F2—C1	1.326 (8)	C8—C9	1.401 (8)
F3—C1	1.345 (7)	C9—H9	0.9500
F4—F5	0.757 (14)	C9—C10	1.382 (7)
F4—F6	1.621 (16)	C10—H10	0.9500
F4—F11	1.751 (18)	C11—H11A	0.9900
F4—F12	1.000 (16)	C11—H11B	0.9900
F4—C14	1.392 (13)	C11—C12	1.522 (8)
F5—F6	0.918 (14)	C12—H12A	0.9900
F5—F7	1.690 (19)	C12—H12B	0.9900
F5—F12	1.544 (18)	C12—C13	1.521 (10)
F5—C14	1.334 (13)	C13—H13A	0.9800
F6—F7	1.017 (15)	C13—H13B	0.9800
F6—F8	1.690 (18)	C13—H13C	0.9800
F6—C14	1.394 (13)	C14—C15	1.535 (7)
F7—F8	0.701 (15)	C15—C16	1.371 (7)
F7—F9	1.278 (17)	C16—H16	0.9500
F7—C14	1.246 (13)	C16—C17	1.430 (7)
F8—F9	0.706 (14)	C17—C18	1.487 (7)
F8—C14	1.360 (14)	C18—C19	1.406 (7)
F9—F10	1.232 (18)	C18—C23	1.393 (7)
F9—F11	1.758 (18)	C19—H19	0.9500
F9—C14	1.347 (14)	C19—C20	1.382 (7)
F10—F11	0.614 (15)	C20—H20	0.9500
F10—F12	1.230 (18)	C20—C21	1.402 (7)
F10—C14	1.371 (15)	C21—C22	1.396 (7)
F11—F12	0.766 (16)	C22—H22	0.9500
F11—C14	1.413 (15)	C22—C23	1.390 (7)
F12—C14	1.218 (13)	C23—H23	0.9500
F13—F14	0.570 (19)	C24—H24A	0.9900
F13—F15	1.004 (17)	C24—H24B	0.9900
F13—F21	1.227 (18)	C24—C25	1.508 (7)
F13—C27	1.275 (12)	C25—H25A	0.9900
F14—F21	1.75 (2)	C25—H25B	0.9900
F14—C27	1.365 (19)	C25—C26	1.522 (9)
F15—F16	1.76 (2)	C26—H26A	0.9800
F15—C27	1.403 (18)	C26—H26B	0.9800
F16—F18	1.049 (18)	C26—H26C	0.9800
F16—C27	1.438 (19)	C27—C28	1.547 (8)
F17—F18	0.670 (18)	C28—C29	1.380 (7)
F17—F19	1.77 (2)	C29—H29	0.9500
F17—C27	1.39 (2)	C29—C30	1.430 (7)
F18—F19	1.167 (17)	C30—C31	1.488 (7)
F18—F20	1.763 (19)	C31—C32	1.403 (7)
F18—C27	1.255 (14)	C31—C36	1.405 (7)
F19—F20	0.637 (15)	C32—H32	0.9500
F19—F21	1.189 (18)	C32—C33	1.395 (7)
F19—C27	1.258 (15)	C33—H33	0.9500
F20—F21	0.748 (15)	C33—C34	1.399 (7)
F20—C27	1.438 (16)	C34—C35	1.396 (7)
F21—C27	1.348 (15)	C35—H35	0.9500
O1—H1	0.8400	C35—C36	1.387 (8)
O1—C40	1.458 (7)	C36—H36	0.9500
O2—C2	1.262 (6)	C37—H37A	0.9900
O3—C8	1.356 (6)	C37—H37B	0.9900
O3—C11	1.442 (8)	C37—C38	1.503 (8)
O4—C17	1.256 (6)	C38—H38A	0.9900
O5—C15	1.271 (6)	C38—H38B	0.9900
O6—C21	1.370 (6)	C38—C39	1.513 (9)
O6—C24	1.442 (6)	C39—H39A	0.9800
O7—C30	1.270 (6)	C39—H39B	0.9800
O8—C28	1.267 (6)	C39—H39C	0.9800
O9—C34	1.365 (6)	C40—H40A	0.9900
O9—C37	1.432 (7)	C40—H40B	0.9900
O10—H10A	0.8624	C40—C41	1.445 (10)
O10—H10B	0.8515	C41—H41A	0.9800
O11—C4	1.263 (6)	C41—H41B	0.9800
C1—C2	1.542 (8)	C41—H41C	0.9800
C2—C3	1.387 (7)		
			
O2—Eu1—O1	139.44 (13)	C9—C10—H10	118.8
O2—Eu1—O10	73.39 (13)	O3—C11—H11A	110.5
O4—Eu1—O1	113.11 (13)	O3—C11—H11B	110.5
O4—Eu1—O2	79.23 (14)	O3—C11—C12	106.3 (5)
O4—Eu1—O5	72.65 (13)	H11A—C11—H11B	108.7
O4—Eu1—O7	142.10 (12)	C12—C11—H11A	110.5
O4—Eu1—O8	75.62 (13)	C12—C11—H11B	110.5
O4—Eu1—O10	144.85 (13)	C11—C12—H12A	109.3
O4—Eu1—O11	78.30 (13)	C11—C12—H12B	109.3
O5—Eu1—O1	69.13 (14)	H12A—C12—H12B	108.0
O5—Eu1—O2	79.32 (14)	C13—C12—C11	111.5 (5)
O5—Eu1—O7	141.35 (12)	C13—C12—H12A	109.3
O5—Eu1—O8	117.36 (14)	C13—C12—H12B	109.3
O5—Eu1—O10	80.86 (15)	C12—C13—H13A	109.5
O5—Eu1—O11	142.03 (12)	C12—C13—H13B	109.5
O7—Eu1—O1	78.45 (13)	C12—C13—H13C	109.5
O7—Eu1—O2	116.35 (14)	H13A—C13—H13B	109.5
O7—Eu1—O8	72.45 (12)	H13A—C13—H13C	109.5
O7—Eu1—O10	71.58 (13)	H13B—C13—H13C	109.5
O8—Eu1—O1	76.23 (14)	F4—C14—F6	71.1 (8)
O8—Eu1—O2	143.05 (13)	F4—C14—F11	77.3 (8)
O8—Eu1—O10	138.49 (14)	F4—C14—C15	110.8 (6)
O10—Eu1—O1	76.91 (14)	F5—C14—F4	32.2 (6)
O11—Eu1—O1	146.94 (13)	F5—C14—F6	39.2 (7)
O11—Eu1—O2	71.59 (13)	F5—C14—F8	111.4 (9)
O11—Eu1—O7	75.15 (12)	F5—C14—F9	130.0 (8)
O11—Eu1—O8	77.09 (14)	F5—C14—F10	123.0 (8)
O11—Eu1—O10	112.36 (15)	F5—C14—F11	105.9 (9)
F5—F4—F6	16.1 (13)	F5—C14—C15	113.8 (7)
F5—F4—F11	116.2 (18)	F6—C14—F11	134.2 (8)
F5—F4—F12	122 (2)	F6—C14—C15	112.9 (6)
F5—F4—C14	69.7 (14)	F7—C14—F4	110.5 (9)
F6—F4—F11	100.1 (9)	F7—C14—F5	81.8 (9)
F12—F4—F6	106.3 (13)	F7—C14—F6	44.9 (7)
F12—F4—F11	6.6 (10)	F7—C14—F8	30.8 (7)
F12—F4—C14	58.5 (10)	F7—C14—F9	58.9 (8)
C14—F4—F6	54.5 (6)	F7—C14—F10	106.9 (9)
C14—F4—F11	51.9 (6)	F7—C14—F11	126.4 (9)
F4—F5—F6	151 (2)	F7—C14—C15	116.0 (7)
F4—F5—F7	119.9 (18)	F8—C14—F4	135.7 (8)
F4—F5—F12	33.2 (13)	F8—C14—F6	75.7 (8)
F4—F5—C14	78.1 (15)	F8—C14—F10	83.5 (9)
F6—F5—F7	30.8 (10)	F8—C14—F11	107.9 (9)
F6—F5—F12	117.6 (15)	F8—C14—C15	108.7 (7)
F6—F5—C14	73.9 (12)	F9—C14—F4	136.7 (8)
F12—F5—F7	86.8 (9)	F9—C14—F6	102.2 (9)
C14—F5—F7	46.9 (6)	F9—C14—F8	30.2 (6)
C14—F5—F12	49.4 (7)	F9—C14—F10	53.9 (8)
F4—F6—F8	100.6 (9)	F9—C14—F11	79.1 (8)
F5—F6—F4	13.3 (11)	F9—C14—C15	110.9 (6)
F5—F6—F7	121.7 (17)	F10—C14—F4	99.6 (8)
F5—F6—F8	113.9 (14)	F10—C14—F6	134.8 (8)
F5—F6—C14	66.8 (11)	F10—C14—F11	25.4 (6)
F7—F6—F4	108.5 (12)	F10—C14—C15	111.7 (7)
F7—F6—F8	8.6 (9)	F11—C14—C15	108.9 (7)
F7—F6—C14	59.8 (9)	F12—C14—F4	44.5 (8)
C14—F6—F4	54.4 (7)	F12—C14—F5	74.3 (9)
C14—F6—F8	51.2 (6)	F12—C14—F6	109.1 (9)
F6—F7—F5	27.5 (9)	F12—C14—F7	129.0 (8)
F6—F7—F9	136.6 (15)	F12—C14—F8	128.7 (9)
F6—F7—C14	75.3 (11)	F12—C14—F9	106.0 (10)
F8—F7—F5	133 (2)	F12—C14—F10	56.4 (8)
F8—F7—F6	159 (2)	F12—C14—F11	32.8 (7)
F8—F7—F9	24.9 (15)	F12—C14—C15	114.8 (7)
F8—F7—C14	83.6 (17)	O5—C15—C14	113.4 (5)
F9—F7—F5	109.1 (11)	O5—C15—C16	129.4 (5)
C14—F7—F5	51.4 (7)	C16—C15—C14	117.1 (5)
C14—F7—F9	64.5 (8)	C15—C16—H16	118.7
F7—F8—F6	12.5 (14)	C15—C16—C17	122.6 (5)
F7—F8—F9	130 (3)	C17—C16—H16	118.7
F7—F8—C14	65.6 (16)	O4—C17—C16	122.3 (5)
F9—F8—F6	120 (2)	O4—C17—C18	117.2 (4)
F9—F8—C14	73.9 (17)	C16—C17—C18	120.5 (4)
C14—F8—F6	53.1 (7)	C19—C18—C17	123.3 (4)
F7—F9—F11	101.8 (11)	C23—C18—C17	118.8 (4)
F7—F9—C14	56.6 (8)	C23—C18—C19	117.9 (5)
F8—F9—F7	24.7 (15)	C18—C19—H19	119.3
F8—F9—F10	138 (2)	C20—C19—C18	121.5 (5)
F8—F9—F11	125 (2)	C20—C19—H19	119.3
F8—F9—C14	75.9 (17)	C19—C20—H20	120.2
F10—F9—F7	113.8 (13)	C19—C20—C21	119.7 (5)
F10—F9—F11	12.4 (9)	C21—C20—H20	120.2
F10—F9—C14	64.0 (9)	O6—C21—C20	116.2 (4)
C14—F9—F11	52.1 (7)	O6—C21—C22	124.1 (5)
F9—F10—C14	62.1 (9)	C22—C21—C20	119.7 (5)
F11—F10—F9	142 (3)	C21—C22—H22	120.2
F11—F10—F12	30.4 (19)	C23—C22—C21	119.7 (5)
F11—F10—C14	81 (2)	C23—C22—H22	120.2
F12—F10—F9	112.6 (14)	C18—C23—H23	119.2
F12—F10—C14	55.5 (8)	C22—C23—C18	121.5 (5)
F4—F11—F9	93.0 (9)	C22—C23—H23	119.2
F10—F11—F4	118 (3)	O6—C24—H24A	110.1
F10—F11—F9	25 (2)	O6—C24—H24B	110.1
F10—F11—F12	126 (3)	O6—C24—C25	108.2 (4)
F10—F11—C14	73 (2)	H24A—C24—H24B	108.4
F12—F11—F4	8.7 (13)	C25—C24—H24A	110.1
F12—F11—F9	101.2 (17)	C25—C24—H24B	110.1
F12—F11—C14	59.5 (14)	C24—C25—H25A	109.4
C14—F11—F4	50.8 (7)	C24—C25—H25B	109.4
C14—F11—F9	48.8 (6)	C24—C25—C26	111.1 (5)
F4—F12—F5	24.5 (9)	H25A—C25—H25B	108.0
F4—F12—F10	141.9 (16)	C26—C25—H25A	109.4
F4—F12—C14	77.0 (11)	C26—C25—H25B	109.4
F10—F12—F5	117.5 (12)	C25—C26—H26A	109.5
F11—F12—F4	165 (2)	C25—C26—H26B	109.5
F11—F12—F5	141.0 (19)	C25—C26—H26C	109.5
F11—F12—F10	23.9 (15)	H26A—C26—H26B	109.5
F11—F12—C14	87.7 (16)	H26A—C26—H26C	109.5
C14—F12—F5	56.3 (7)	H26B—C26—H26C	109.5
C14—F12—F10	68.1 (9)	F13—C27—F14	24.6 (8)
F14—F13—F15	14 (3)	F13—C27—F15	43.7 (8)
F14—F13—F21	151 (3)	F13—C27—F16	109.9 (11)
F14—F13—C27	87 (2)	F13—C27—F17	117.1 (11)
F15—F13—F21	140.1 (15)	F13—C27—F20	85.4 (9)
F15—F13—C27	74.9 (11)	F13—C27—F21	55.7 (8)
F21—F13—C27	65.2 (9)	F13—C27—C28	119.0 (8)
F13—F14—F21	20 (2)	F14—C27—F15	19.6 (8)
F13—F14—C27	69 (2)	F14—C27—F16	93.4 (10)
C27—F14—F21	49.5 (7)	F14—C27—F17	105.4 (11)
F13—F15—F16	103.6 (14)	F14—C27—F20	110.0 (9)
F13—F15—C27	61.3 (10)	F14—C27—C28	110.9 (9)
C27—F15—F16	52.6 (8)	F15—C27—F16	76.5 (9)
F18—F16—F15	101.9 (14)	F15—C27—F20	128.1 (9)
F18—F16—C27	58.1 (10)	F15—C27—C28	106.9 (8)
C27—F16—F15	50.8 (8)	F16—C27—C28	109.3 (9)
F18—F17—F19	20.7 (19)	F17—C27—F15	90.6 (10)
F18—F17—C27	64 (2)	F17—C27—F16	16.4 (9)
C27—F17—F19	45.0 (7)	F17—C27—F20	109.6 (9)
F16—F18—F19	137.5 (16)	F17—C27—C28	113.7 (9)
F16—F18—F20	128.5 (13)	F18—C27—F13	122.4 (10)
F16—F18—C27	76.7 (12)	F18—C27—F14	122.9 (11)
F17—F18—F16	10 (3)	F18—C27—F15	113.7 (10)
F17—F18—F19	148 (3)	F18—C27—F16	45.2 (8)
F17—F18—F20	139 (3)	F18—C27—F17	28.8 (8)
F17—F18—C27	87 (2)	F18—C27—F19	55.4 (8)
F19—F18—F20	9.0 (10)	F18—C27—F20	81.5 (9)
F19—F18—C27	62.4 (10)	F18—C27—F21	104.8 (10)
C27—F18—F20	53.8 (8)	F18—C27—C28	118.5 (8)
F18—F19—F17	11.7 (10)	F19—C27—F13	101.8 (10)
F18—F19—F21	122.3 (16)	F19—C27—F14	123.9 (10)
F18—F19—C27	62.2 (10)	F19—C27—F15	136.1 (10)
F20—F19—F17	143 (3)	F19—C27—F16	99.9 (9)
F20—F19—F18	154 (3)	F19—C27—F17	83.8 (9)
F20—F19—F21	33.7 (19)	F19—C27—F20	26.3 (7)
F20—F19—C27	93 (2)	F19—C27—F21	54.2 (8)
F21—F19—F17	110.8 (13)	F19—C27—C28	115.2 (8)
F21—F19—C27	66.8 (10)	F20—C27—F16	125.1 (9)
C27—F19—F17	51.2 (8)	F20—C27—C28	107.2 (7)
F19—F20—F18	16.6 (19)	F21—C27—F14	80.2 (9)
F19—F20—F21	118 (3)	F21—C27—F15	99.4 (9)
F19—F20—C27	61 (2)	F21—C27—F16	137.9 (10)
F21—F20—F18	103 (2)	F21—C27—F17	127.8 (10)
F21—F20—C27	67.9 (17)	F21—C27—F20	30.9 (6)
C27—F20—F18	44.7 (6)	F21—C27—C28	111.9 (7)
F13—F21—F14	9.0 (9)	O8—C28—C27	112.7 (5)
F13—F21—C27	59.1 (9)	O8—C28—C29	129.5 (5)
F19—F21—F13	108.9 (14)	C29—C28—C27	117.8 (5)
F19—F21—F14	102.3 (12)	C28—C29—H29	118.2
F19—F21—C27	59.0 (9)	C28—C29—C30	123.7 (5)
F20—F21—F13	137 (2)	C30—C29—H29	118.2
F20—F21—F14	129 (2)	O7—C30—C29	121.9 (5)
F20—F21—F19	28.2 (15)	O7—C30—C31	117.2 (4)
F20—F21—C27	81.2 (17)	C29—C30—C31	121.0 (4)
C27—F21—F14	50.3 (7)	C32—C31—C30	123.8 (4)
Eu1—O1—H1	106.2	C32—C31—C36	118.0 (5)
C40—O1—Eu1	129.4 (3)	C36—C31—C30	118.1 (4)
C40—O1—H1	109.5	C31—C32—H32	119.5
C2—O2—Eu1	129.0 (3)	C33—C32—C31	120.9 (5)
C8—O3—C11	118.6 (5)	C33—C32—H32	119.5
C17—O4—Eu1	138.7 (3)	C32—C33—H33	120.1
C15—O5—Eu1	132.2 (3)	C32—C33—C34	119.8 (5)
C21—O6—C24	117.8 (4)	C34—C33—H33	120.1
C30—O7—Eu1	135.1 (3)	O9—C34—C33	124.0 (5)
C28—O8—Eu1	129.3 (3)	O9—C34—C35	115.9 (5)
C34—O9—C37	119.3 (4)	C35—C34—C33	120.1 (5)
Eu1—O10—H10A	127.4	C34—C35—H35	120.2
Eu1—O10—H10B	127.8	C36—C35—C34	119.5 (5)
H10A—O10—H10B	104.8	C36—C35—H35	120.2
C4—O11—Eu1	134.8 (3)	C31—C36—H36	119.2
F1—C1—F3	105.7 (6)	C35—C36—C31	121.6 (5)
F1—C1—C2	111.7 (5)	C35—C36—H36	119.2
F2—C1—F1	106.7 (5)	O9—C37—H37A	110.2
F2—C1—F3	107.7 (5)	O9—C37—H37B	110.2
F2—C1—C2	111.5 (6)	O9—C37—C38	107.4 (5)
F3—C1—C2	113.1 (5)	H37A—C37—H37B	108.5
O2—C2—C1	113.3 (5)	C38—C37—H37A	110.2
O2—C2—C3	129.2 (5)	C38—C37—H37B	110.2
C3—C2—C1	117.4 (5)	C37—C38—H38A	109.0
C2—C3—H3	118.9	C37—C38—H38B	109.0
C2—C3—C4	122.2 (5)	C37—C38—C39	112.8 (6)
C4—C3—H3	118.9	H38A—C38—H38B	107.8
O11—C4—C3	122.7 (5)	C39—C38—H38A	109.0
O11—C4—C5	117.3 (5)	C39—C38—H38B	109.0
C3—C4—C5	120.0 (5)	C38—C39—H39A	109.5
C6—C5—C4	123.0 (5)	C38—C39—H39B	109.5
C10—C5—C4	118.8 (5)	C38—C39—H39C	109.5
C10—C5—C6	118.2 (5)	H39A—C39—H39B	109.5
C5—C6—H6	119.6	H39A—C39—H39C	109.5
C7—C6—C5	120.7 (5)	H39B—C39—H39C	109.5
C7—C6—H6	119.6	O1—C40—H40A	109.3
C6—C7—H7	120.1	O1—C40—H40B	109.3
C6—C7—C8	119.9 (5)	H40A—C40—H40B	107.9
C8—C7—H7	120.1	C41—C40—O1	111.8 (6)
O3—C8—C7	115.4 (5)	C41—C40—H40A	109.3
O3—C8—C9	124.5 (5)	C41—C40—H40B	109.3
C9—C8—C7	120.1 (5)	C40—C41—H41A	109.5
C8—C9—H9	120.6	C40—C41—H41B	109.5
C10—C9—C8	118.7 (5)	C40—C41—H41C	109.5
C10—C9—H9	120.6	H41A—C41—H41B	109.5
C5—C10—H10	118.8	H41A—C41—H41C	109.5
C9—C10—C5	122.3 (5)	H41B—C41—H41C	109.5
			
Eu1—O1—C40—C41	−128.6 (6)	F12—F10—C14—F11	17.8 (18)
Eu1—O2—C2—C1	−156.8 (4)	F12—F10—C14—C15	106.1 (9)
Eu1—O2—C2—C3	25.9 (9)	F12—F11—C14—F4	−1.1 (14)
Eu1—O4—C17—C16	5.6 (8)	F12—F11—C14—F5	−15.9 (16)
Eu1—O4—C17—C18	−172.6 (3)	F12—F11—C14—F6	−48.1 (19)
Eu1—O5—C15—C14	164.8 (4)	F12—F11—C14—F7	−107.4 (17)
Eu1—O5—C15—C16	−14.1 (9)	F12—F11—C14—F8	−135.4 (15)
Eu1—O7—C30—C29	21.0 (8)	F12—F11—C14—F9	−144.6 (15)
Eu1—O7—C30—C31	−160.6 (3)	F12—F11—C14—F10	−152 (3)
Eu1—O8—C28—C27	160.7 (4)	F12—F11—C14—C15	106.8 (14)
Eu1—O8—C28—C29	−21.3 (9)	F12—C14—C15—O5	73.5 (11)
Eu1—O11—C4—C3	−21.5 (8)	F12—C14—C15—C16	−107.4 (11)
Eu1—O11—C4—C5	159.2 (3)	F13—F14—F21—F19	−138 (7)
F1—C1—C2—O2	42.1 (8)	F13—F14—F21—F20	−147 (7)
F1—C1—C2—C3	−140.2 (6)	F13—F14—F21—C27	−168 (7)
F2—C1—C2—O2	−77.2 (7)	F13—F14—C27—F15	−164 (6)
F2—C1—C2—C3	100.5 (6)	F13—F14—C27—F16	−134 (3)
F3—C1—C2—O2	161.2 (6)	F13—F14—C27—F17	−122 (3)
F3—C1—C2—C3	−21.1 (9)	F13—F14—C27—F18	−97 (3)
F4—F5—F6—F7	6 (5)	F13—F14—C27—F19	−29 (3)
F4—F5—F6—F8	2 (4)	F13—F14—C27—F20	−4 (3)
F4—F5—F6—C14	−19 (4)	F13—F14—C27—F21	4 (3)
F4—F5—F7—F6	−176 (3)	F13—F14—C27—C28	114 (3)
F4—F5—F7—F8	−9 (3)	F13—F15—F16—F18	6.5 (17)
F4—F5—F7—F9	0.5 (18)	F13—F15—F16—C27	36.2 (11)
F4—F5—F7—C14	−30.2 (15)	F13—F15—C27—F14	10 (4)
F4—F5—F12—F10	−176 (2)	F13—F15—C27—F16	−139.1 (13)
F4—F5—F12—F11	−170 (3)	F13—F15—C27—F17	−130.4 (13)
F4—F5—F12—C14	−144 (2)	F13—F15—C27—F18	−112.8 (13)
F4—F5—C14—F6	170.8 (19)	F13—F15—C27—F19	−48.8 (18)
F4—F5—C14—F7	153.6 (14)	F13—F15—C27—F20	−14.7 (15)
F4—F5—C14—F8	145.0 (13)	F13—F15—C27—F21	−1.9 (12)
F4—F5—C14—F9	116.9 (16)	F13—F15—C27—C28	114.5 (11)
F4—F5—C14—F10	48.7 (17)	F13—F21—C27—F14	−2.3 (14)
F4—F5—C14—F11	27.9 (14)	F13—F21—C27—F15	1.6 (10)
F4—F5—C14—F12	19.0 (13)	F13—F21—C27—F16	81.9 (17)
F4—F5—C14—C15	−91.6 (13)	F13—F21—C27—F17	99.7 (15)
F4—F6—F7—F5	1.6 (11)	F13—F21—C27—F18	119.4 (12)
F4—F6—F7—F8	−24 (6)	F13—F21—C27—F19	143.0 (13)
F4—F6—F7—F9	−3 (2)	F13—F21—C27—F20	162 (2)
F4—F6—F7—C14	−25.1 (8)	F13—F21—C27—C28	−111.0 (10)
F4—F6—F8—F7	156 (6)	F13—C27—C28—O8	−13.1 (12)
F4—F6—F8—F9	9 (2)	F13—C27—C28—C29	168.6 (10)
F4—F6—F8—C14	−24.3 (6)	F14—F13—F15—F16	−179 (100)
F4—F6—C14—F5	−5.2 (11)	F14—F13—F15—C27	−146 (12)
F4—F6—C14—F7	150.3 (11)	F14—F13—F21—F19	44 (7)
F4—F6—C14—F8	150.1 (8)	F14—F13—F21—F20	38 (8)
F4—F6—C14—F9	135.5 (8)	F14—F13—F21—C27	11 (7)
F4—F6—C14—F10	84.8 (13)	F14—F13—C27—F15	8 (3)
F4—F6—C14—F11	48.9 (12)	F14—F13—C27—F16	50 (3)
F4—F6—C14—F12	23.6 (9)	F14—F13—C27—F17	66 (3)
F4—F6—C14—C15	−105.3 (7)	F14—F13—C27—F18	99 (3)
F4—F11—F12—F5	16 (6)	F14—F13—C27—F19	155 (3)
F4—F11—F12—F10	28 (9)	F14—F13—C27—F20	176 (3)
F4—F11—F12—C14	−6 (7)	F14—F13—C27—F21	−175 (3)
F4—F11—C14—F5	−14.8 (8)	F14—F13—C27—C28	−77 (3)
F4—F11—C14—F6	−47.0 (12)	F14—F21—C27—F13	2.3 (14)
F4—F11—C14—F7	−106.3 (12)	F14—F21—C27—F15	3.9 (13)
F4—F11—C14—F8	−134.3 (9)	F14—F21—C27—F16	84.2 (16)
F4—F11—C14—F9	−143.5 (7)	F14—F21—C27—F17	102.0 (14)
F4—F11—C14—F10	−151 (2)	F14—F21—C27—F18	121.6 (11)
F4—F11—C14—F12	1.1 (14)	F14—F21—C27—F19	145.3 (12)
F4—F11—C14—C15	107.9 (7)	F14—F21—C27—F20	164.1 (17)
F4—F12—C14—F5	−14.4 (10)	F14—F21—C27—C28	−108.7 (10)
F4—F12—C14—F6	−32.8 (11)	F14—C27—C28—O8	−38.9 (10)
F4—F12—C14—F7	−79.6 (16)	F14—C27—C28—C29	142.9 (8)
F4—F12—C14—F8	−119.5 (13)	F15—F13—F14—F21	43 (16)
F4—F12—C14—F9	−142.2 (10)	F15—F13—F14—C27	32 (11)
F4—F12—C14—F10	−164.5 (12)	F15—F13—F21—F14	−15 (6)
F4—F12—C14—F11	−178.5 (19)	F15—F13—F21—F19	30 (3)
F4—F12—C14—C15	95.0 (10)	F15—F13—F21—F20	23 (4)
F4—C14—C15—O5	121.8 (8)	F15—F13—F21—C27	−3 (2)
F4—C14—C15—C16	−59.2 (9)	F15—F13—C27—F14	−8 (3)
F5—F4—F6—F7	−174 (4)	F15—F13—C27—F16	42.6 (13)
F5—F4—F6—F8	−178 (4)	F15—F13—C27—F17	58.8 (14)
F5—F4—F6—C14	159 (4)	F15—F13—C27—F18	91.5 (15)
F5—F4—F11—F9	2.2 (16)	F15—F13—C27—F19	147.8 (12)
F5—F4—F11—F10	−3 (3)	F15—F13—C27—F20	168.4 (12)
F5—F4—F11—F12	−157 (8)	F15—F13—C27—F21	177.7 (14)
F5—F4—F11—C14	28.9 (14)	F15—F13—C27—C28	−84.4 (12)
F5—F4—F12—F10	6 (3)	F15—F16—F18—F17	−162 (16)
F5—F4—F12—F11	24 (8)	F15—F16—F18—F19	11 (3)
F5—F4—F12—C14	29.8 (18)	F15—F16—F18—F20	11.9 (18)
F5—F4—C14—F6	−6.1 (13)	F15—F16—F18—C27	26.9 (9)
F5—F4—C14—F7	−28.1 (15)	F15—F16—C27—F13	−28.8 (9)
F5—F4—C14—F8	−49.8 (19)	F15—F16—C27—F14	−10.0 (12)
F5—F4—C14—F9	−94.0 (18)	F15—F16—C27—F17	−148 (5)
F5—F4—C14—F10	−140.3 (14)	F15—F16—C27—F18	−145.2 (12)
F5—F4—C14—F11	−152.5 (14)	F15—F16—C27—F19	−135.3 (10)
F5—F4—C14—F12	−153.3 (18)	F15—F16—C27—F20	−127.4 (12)
F5—F4—C14—C15	101.9 (13)	F15—F16—C27—F21	−89.2 (15)
F5—F6—F7—F8	−26 (7)	F15—F16—C27—C28	103.5 (9)
F5—F6—F7—F9	−4 (3)	F15—C27—C28—O8	−59.1 (9)
F5—F6—F7—C14	−26.7 (15)	F15—C27—C28—C29	122.6 (8)
F5—F6—F8—F7	156 (6)	F16—F15—C27—F13	139.1 (13)
F5—F6—F8—F9	8 (2)	F16—F15—C27—F14	149 (4)
F5—F6—F8—C14	−24.9 (11)	F16—F15—C27—F17	8.7 (13)
F5—F6—C14—F4	5.2 (11)	F16—F15—C27—F18	26.3 (10)
F5—F6—C14—F7	155.4 (15)	F16—F15—C27—F19	90.3 (15)
F5—F6—C14—F8	155.3 (11)	F16—F15—C27—F20	124.4 (12)
F5—F6—C14—F9	140.7 (11)	F16—F15—C27—F21	137.2 (10)
F5—F6—C14—F10	90.0 (16)	F16—F15—C27—C28	−106.4 (9)
F5—F6—C14—F11	54.1 (16)	F16—F18—F19—F17	−2 (5)
F5—F6—C14—F12	28.8 (12)	F16—F18—F19—F20	3 (7)
F5—F6—C14—C15	−100.1 (10)	F16—F18—F19—F21	−14 (3)
F5—F7—F8—F6	−16 (4)	F16—F18—F19—C27	17 (2)
F5—F7—F8—F9	21 (4)	F16—F18—F20—F19	−177 (6)
F5—F7—F8—C14	−16.7 (16)	F16—F18—F20—F21	−21 (2)
F5—F7—F9—F8	−164 (3)	F16—F18—F20—C27	18.3 (15)
F5—F7—F9—F10	3.9 (13)	F16—F18—C27—F13	−86.4 (15)
F5—F7—F9—F11	1.1 (10)	F16—F18—C27—F14	−57.1 (15)
F5—F7—F9—C14	−26.2 (6)	F16—F18—C27—F15	−37.4 (13)
F5—F7—C14—F4	14.7 (8)	F16—F18—C27—F17	2 (3)
F5—F7—C14—F6	−15.4 (9)	F16—F18—C27—F19	−168.1 (15)
F5—F7—C14—F8	164.3 (16)	F16—F18—C27—F20	−165.4 (12)
F5—F7—C14—F9	147.7 (9)	F16—F18—C27—F21	−144.9 (12)
F5—F7—C14—F10	122.1 (9)	F16—F18—C27—C28	89.5 (13)
F5—F7—C14—F11	103.8 (12)	F16—C27—C28—O8	−140.5 (8)
F5—F7—C14—F12	62.0 (14)	F16—C27—C28—C29	41.3 (10)
F5—F7—C14—C15	−112.5 (8)	F17—F18—F19—F20	5 (9)
F5—F12—C14—F4	14.4 (10)	F17—F18—F19—F21	−11 (6)
F5—F12—C14—F6	−18.5 (8)	F17—F18—F19—C27	19 (5)
F5—F12—C14—F7	−65.2 (14)	F17—F18—F20—F19	−176 (7)
F5—F12—C14—F8	−105.1 (13)	F17—F18—F20—F21	−19 (4)
F5—F12—C14—F9	−127.8 (9)	F17—F18—F20—C27	20 (4)
F5—F12—C14—F10	−150.1 (9)	F17—F18—C27—F13	−88 (3)
F5—F12—C14—F11	−164.1 (15)	F17—F18—C27—F14	−59 (3)
F5—F12—C14—C15	109.4 (8)	F17—F18—C27—F15	−39 (3)
F5—C14—C15—O5	156.5 (9)	F17—F18—C27—F16	−2 (3)
F5—C14—C15—C16	−24.5 (11)	F17—F18—C27—F19	−170 (3)
F6—F4—F5—F7	4 (3)	F17—F18—C27—F20	−167 (2)
F6—F4—F5—F12	9 (5)	F17—F18—C27—F21	−147 (2)
F6—F4—F5—C14	−18 (3)	F17—F18—C27—C28	88 (2)
F6—F4—F11—F9	0.6 (8)	F17—F19—F20—F18	2 (3)
F6—F4—F11—F10	−5 (2)	F17—F19—F20—F21	−24 (5)
F6—F4—F11—F12	−159 (8)	F17—F19—F20—C27	14 (3)
F6—F4—F11—C14	27.3 (6)	F17—F19—F21—F13	−6.1 (15)
F6—F4—F12—F5	−2.5 (14)	F17—F19—F21—F14	0.4 (13)
F6—F4—F12—F10	4 (2)	F17—F19—F21—F20	165 (3)
F6—F4—F12—F11	22 (8)	F17—F19—F21—C27	27.0 (9)
F6—F4—F12—C14	27.3 (8)	F17—F19—C27—F13	−116.5 (12)
F6—F4—C14—F5	6.1 (13)	F17—F19—C27—F14	−104.4 (14)
F6—F4—C14—F7	−21.9 (8)	F17—F19—C27—F15	−84.4 (15)
F6—F4—C14—F8	−43.7 (13)	F17—F19—C27—F16	−3.6 (14)
F6—F4—C14—F9	−87.9 (13)	F17—F19—C27—F18	5.0 (13)
F6—F4—C14—F10	−134.2 (8)	F17—F19—C27—F20	−169 (2)
F6—F4—C14—F11	−146.4 (8)	F17—F19—C27—F21	−147.0 (12)
F6—F4—C14—F12	−147.2 (11)	F17—F19—C27—C28	113.3 (10)
F6—F4—C14—C15	108.0 (7)	F17—C27—C28—O8	−157.5 (8)
F6—F5—F7—F8	168 (3)	F17—C27—C28—C29	24.3 (11)
F6—F5—F7—F9	176.9 (19)	F18—F16—C27—F13	116.4 (13)
F6—F5—F7—C14	146.2 (19)	F18—F16—C27—F14	135.1 (12)
F6—F5—F12—F4	175 (3)	F18—F16—C27—F15	145.2 (12)
F6—F5—F12—F10	−0.6 (17)	F18—F16—C27—F17	−3 (5)
F6—F5—F12—F11	5 (3)	F18—F16—C27—F19	9.9 (12)
F6—F5—F12—C14	30.9 (12)	F18—F16—C27—F20	17.7 (15)
F6—F5—C14—F4	−170.8 (19)	F18—F16—C27—F21	55.9 (18)
F6—F5—C14—F7	−17.3 (10)	F18—F16—C27—C28	−111.3 (11)
F6—F5—C14—F8	−25.8 (12)	F18—F17—F19—F20	−176 (7)
F6—F5—C14—F9	−53.9 (15)	F18—F17—F19—F21	170 (5)
F6—F5—C14—F10	−122.1 (12)	F18—F17—F19—C27	−158 (6)
F6—F5—C14—F11	−142.9 (11)	F18—F17—C27—F13	109 (2)
F6—F5—C14—F12	−151.8 (12)	F18—F17—C27—F14	132 (2)
F6—F5—C14—C15	97.6 (10)	F18—F17—C27—F15	145 (2)
F6—F7—F8—F9	37 (8)	F18—F17—C27—F16	176 (7)
F6—F7—F8—C14	−1 (6)	F18—F17—C27—F19	8 (2)
F6—F7—F9—F8	−161 (4)	F18—F17—C27—F20	14 (3)
F6—F7—F9—F10	6 (2)	F18—F17—C27—F21	42 (3)
F6—F7—F9—F11	3.1 (19)	F18—F17—C27—C28	−106 (2)
F6—F7—F9—C14	−24.1 (15)	F18—F19—F20—F21	−26 (8)
F6—F7—C14—F4	30.1 (11)	F18—F19—F20—C27	12 (5)
F6—F7—C14—F5	15.4 (9)	F18—F19—F21—F13	−4 (2)
F6—F7—C14—F8	180 (2)	F18—F19—F21—F14	2.8 (17)
F6—F7—C14—F9	163.1 (12)	F18—F19—F21—F20	167 (4)
F6—F7—C14—F10	137.5 (10)	F18—F19—F21—C27	29.5 (13)
F6—F7—C14—F11	119.2 (12)	F18—F19—C27—F13	−121.4 (12)
F6—F7—C14—F12	77.4 (16)	F18—F19—C27—F14	−109.4 (13)
F6—F7—C14—C15	−97.1 (10)	F18—F19—C27—F15	−89.3 (16)
F6—F8—F9—F7	8.8 (19)	F18—F19—C27—F16	−8.5 (11)
F6—F8—F9—F10	−8 (3)	F18—F19—C27—F17	−5.0 (13)
F6—F8—F9—F11	−10 (3)	F18—F19—C27—F20	−174 (3)
F6—F8—F9—C14	−27.0 (12)	F18—F19—C27—F21	−151.9 (13)
F6—F8—C14—F4	42.4 (12)	F18—F19—C27—C28	108.4 (10)
F6—F8—C14—F5	16.5 (8)	F18—F20—F21—F13	5 (3)
F6—F8—C14—F7	−0.2 (15)	F18—F20—F21—F14	13 (2)
F6—F8—C14—F9	150.6 (16)	F18—F20—F21—F19	−7 (2)
F6—F8—C14—F10	139.5 (8)	F18—F20—F21—C27	28.4 (10)
F6—F8—C14—F11	132.4 (8)	F18—F20—C27—F13	−123.8 (10)
F6—F8—C14—F12	103.2 (13)	F18—F20—C27—F14	−122.0 (11)
F6—F8—C14—C15	−109.7 (7)	F18—F20—C27—F15	−113.6 (13)
F6—C14—C15—O5	−160.6 (8)	F18—F20—C27—F16	−12.6 (11)
F6—C14—C15—C16	18.4 (10)	F18—F20—C27—F17	−6.6 (12)
F7—F5—F6—F4	−6 (5)	F18—F20—C27—F19	5 (2)
F7—F5—F6—F8	−4.1 (10)	F18—F20—C27—F21	−138.8 (17)
F7—F5—F6—C14	−25.0 (14)	F18—F20—C27—C28	117.3 (8)
F7—F5—F12—F4	176 (2)	F18—C27—C28—O8	170.8 (10)
F7—F5—F12—F10	0.0 (11)	F18—C27—C28—C29	−7.5 (12)
F7—F5—F12—F11	6 (3)	F19—F17—F18—F16	8 (19)
F7—F5—F12—C14	31.4 (6)	F19—F17—F18—F20	1 (2)
F7—F5—C14—F4	−153.6 (14)	F19—F17—F18—C27	17 (4)
F7—F5—C14—F6	17.3 (10)	F19—F17—C27—F13	100.1 (12)
F7—F5—C14—F8	−8.5 (9)	F19—F17—C27—F14	123.5 (11)
F7—F5—C14—F9	−36.7 (11)	F19—F17—C27—F15	136.4 (10)
F7—F5—C14—F10	−104.8 (11)	F19—F17—C27—F16	167 (5)
F7—F5—C14—F11	−125.6 (9)	F19—F17—C27—F18	−8 (2)
F7—F5—C14—F12	−134.5 (9)	F19—F17—C27—F20	5.2 (10)
F7—F5—C14—C15	114.8 (8)	F19—F17—C27—F21	34.0 (12)
F7—F6—F8—F9	−148 (7)	F19—F17—C27—C28	−114.8 (9)
F7—F6—F8—C14	179 (6)	F19—F18—F20—F21	157 (7)
F7—F6—C14—F4	−150.3 (11)	F19—F18—F20—C27	−164 (6)
F7—F6—C14—F5	−155.4 (15)	F19—F18—C27—F13	81.7 (14)
F7—F6—C14—F8	−0.1 (11)	F19—F18—C27—F14	111.1 (13)
F7—F6—C14—F9	−14.8 (11)	F19—F18—C27—F15	130.8 (12)
F7—F6—C14—F10	−65.4 (15)	F19—F18—C27—F16	168.1 (15)
F7—F6—C14—F11	−101.4 (14)	F19—F18—C27—F17	170 (3)
F7—F6—C14—F12	−126.6 (12)	F19—F18—C27—F20	2.7 (11)
F7—F6—C14—C15	104.5 (10)	F19—F18—C27—F21	23.2 (11)
F7—F8—F9—F10	−17 (5)	F19—F18—C27—C28	−102.3 (11)
F7—F8—F9—F11	−19 (4)	F19—F20—F21—F13	13 (5)
F7—F8—F9—C14	−36 (3)	F19—F20—F21—F14	20 (4)
F7—F8—C14—F4	43 (2)	F19—F20—F21—C27	36 (3)
F7—F8—C14—F5	16.7 (17)	F19—F20—C27—F13	−129 (2)
F7—F8—C14—F6	0.2 (15)	F19—F20—C27—F14	−127 (2)
F7—F8—C14—F9	151 (2)	F19—F20—C27—F15	−119 (2)
F7—F8—C14—F10	139.7 (16)	F19—F20—C27—F16	−18 (3)
F7—F8—C14—F11	132.5 (16)	F19—F20—C27—F17	−12 (2)
F7—F8—C14—F12	103.3 (19)	F19—F20—C27—F18	−5 (2)
F7—F8—C14—C15	−109.5 (15)	F19—F20—C27—F21	−144 (3)
F7—F9—F10—F11	−13 (4)	F19—F20—C27—C28	112 (2)
F7—F9—F10—F12	−4.0 (15)	F19—F21—C27—F13	−143.0 (13)
F7—F9—F10—C14	−27.8 (9)	F19—F21—C27—F14	−145.3 (12)
F7—F9—F11—F4	−1.5 (9)	F19—F21—C27—F15	−141.4 (11)
F7—F9—F11—F10	168 (4)	F19—F21—C27—F16	−61.1 (17)
F7—F9—F11—F12	1.5 (16)	F19—F21—C27—F17	−43.3 (15)
F7—F9—F11—C14	−29.0 (7)	F19—F21—C27—F18	−23.6 (11)
F7—F9—C14—F4	87.5 (15)	F19—F21—C27—F20	18.8 (16)
F7—F9—C14—F5	43.6 (13)	F19—F21—C27—C28	106.0 (10)
F7—F9—C14—F6	12.1 (9)	F19—C27—C28—O8	108.1 (9)
F7—F9—C14—F8	−17.0 (15)	F19—C27—C28—C29	−70.1 (10)
F7—F9—C14—F10	149.3 (11)	F20—F18—F19—F17	−5 (9)
F7—F9—C14—F11	145.3 (9)	F20—F18—F19—F21	−17 (5)
F7—F9—C14—F12	126.3 (10)	F20—F18—F19—C27	14 (6)
F7—F9—C14—C15	−108.5 (8)	F20—F18—C27—F13	79.0 (12)
F7—C14—C15—O5	−111.2 (10)	F20—F18—C27—F14	108.4 (12)
F7—C14—C15—C16	67.9 (11)	F20—F18—C27—F15	128.0 (11)
F8—F6—F7—F5	26 (7)	F20—F18—C27—F16	165.4 (12)
F8—F6—F7—F9	22 (5)	F20—F18—C27—F17	167 (2)
F8—F6—F7—C14	−1 (6)	F20—F18—C27—F19	−2.7 (11)
F8—F6—C14—F4	−150.1 (8)	F20—F18—C27—F21	20.5 (8)
F8—F6—C14—F5	−155.3 (11)	F20—F18—C27—C28	−105.1 (9)
F8—F6—C14—F7	0.1 (11)	F20—F19—F21—F13	−171 (4)
F8—F6—C14—F9	−14.6 (8)	F20—F19—F21—F14	−164 (3)
F8—F6—C14—F10	−65.3 (13)	F20—F19—F21—C27	−138 (3)
F8—F6—C14—F11	−101.2 (13)	F20—F19—C27—F13	53 (2)
F8—F6—C14—F12	−126.5 (10)	F20—F19—C27—F14	65 (2)
F8—F6—C14—C15	104.6 (8)	F20—F19—C27—F15	85 (2)
F8—F7—F9—F10	167 (4)	F20—F19—C27—F16	165 (2)
F8—F7—F9—F11	165 (3)	F20—F19—C27—F17	169 (2)
F8—F7—F9—C14	137 (3)	F20—F19—C27—F18	174 (3)
F8—F7—C14—F4	−149.7 (15)	F20—F19—C27—F21	22.0 (19)
F8—F7—C14—F5	−164.3 (16)	F20—F19—C27—C28	−78 (2)
F8—F7—C14—F6	−180 (2)	F20—F21—C27—F13	−162 (2)
F8—F7—C14—F9	−16.7 (14)	F20—F21—C27—F14	−164.1 (17)
F8—F7—C14—F10	−42.2 (17)	F20—F21—C27—F15	−160.2 (16)
F8—F7—C14—F11	−60.6 (19)	F20—F21—C27—F16	−80 (2)
F8—F7—C14—F12	−102.3 (19)	F20—F21—C27—F17	−62 (2)
F8—F7—C14—C15	83.1 (16)	F20—F21—C27—F18	−42.4 (17)
F8—F9—F10—F11	−5 (5)	F20—F21—C27—F19	−18.8 (16)
F8—F9—F10—F12	4 (3)	F20—F21—C27—C28	87.2 (16)
F8—F9—F10—C14	−20 (3)	F20—C27—C28—O8	81.2 (9)
F8—F9—F11—F4	6 (2)	F20—C27—C28—C29	−97.0 (9)
F8—F9—F11—F10	176 (4)	F21—F13—F14—C27	−10 (6)
F8—F9—F11—F12	9 (2)	F21—F13—F15—F16	−29 (3)
F8—F9—F11—C14	−21.2 (18)	F21—F13—F15—C27	3 (2)
F8—F9—C14—F4	104.5 (18)	F21—F13—C27—F14	175 (3)
F8—F9—C14—F5	60.6 (19)	F21—F13—C27—F15	−177.7 (14)
F8—F9—C14—F6	29.1 (16)	F21—F13—C27—F16	−135.0 (11)
F8—F9—C14—F7	17.0 (15)	F21—F13—C27—F17	−118.9 (12)
F8—F9—C14—F10	166.2 (18)	F21—F13—C27—F18	−86.2 (14)
F8—F9—C14—F11	162.3 (16)	F21—F13—C27—F19	−29.9 (11)
F8—F9—C14—F12	143.3 (15)	F21—F13—C27—F20	−9.3 (10)
F8—F9—C14—C15	−91.5 (15)	F21—F13—C27—C28	97.9 (10)
F8—C14—C15—O5	−78.7 (9)	F21—F14—C27—F13	−4 (3)
F8—C14—C15—C16	100.4 (9)	F21—F14—C27—F15	−169 (4)
F9—F7—F8—F6	−37 (8)	F21—F14—C27—F16	−138.0 (10)
F9—F7—F8—C14	−38 (3)	F21—F14—C27—F17	−126.7 (10)
F9—F7—C14—F4	−133.0 (9)	F21—F14—C27—F18	−101.4 (12)
F9—F7—C14—F5	−147.7 (9)	F21—F14—C27—F19	−33.8 (11)
F9—F7—C14—F6	−163.1 (12)	F21—F14—C27—F20	−8.6 (9)
F9—F7—C14—F8	16.7 (14)	F21—F14—C27—C28	109.8 (8)
F9—F7—C14—F10	−25.6 (9)	F21—F19—F20—F18	26 (8)
F9—F7—C14—F11	−43.9 (13)	F21—F19—F20—C27	38 (3)
F9—F7—C14—F12	−85.7 (15)	F21—F19—C27—F13	30.5 (11)
F9—F7—C14—C15	99.8 (8)	F21—F19—C27—F14	42.6 (14)
F9—F8—C14—F4	−108.2 (18)	F21—F19—C27—F15	62.6 (16)
F9—F8—C14—F5	−134.1 (15)	F21—F19—C27—F16	143.4 (11)
F9—F8—C14—F6	−150.6 (16)	F21—F19—C27—F17	147.0 (12)
F9—F8—C14—F7	−151 (2)	F21—F19—C27—F18	151.9 (13)
F9—F8—C14—F10	−11.2 (15)	F21—F19—C27—F20	−22.0 (19)
F9—F8—C14—F11	−18.3 (17)	F21—F19—C27—C28	−99.7 (9)
F9—F8—C14—F12	−47 (2)	F21—F20—C27—F13	15.0 (16)
F9—F8—C14—C15	99.6 (15)	F21—F20—C27—F14	16.7 (18)
F9—F10—F11—F4	12 (5)	F21—F20—C27—F15	25 (2)
F9—F10—F11—F12	17 (6)	F21—F20—C27—F16	126.1 (17)
F9—F10—F11—C14	−13 (3)	F21—F20—C27—F17	132.2 (17)
F9—F10—F12—F4	−1 (2)	F21—F20—C27—F18	138.8 (17)
F9—F10—F12—F5	2.3 (15)	F21—F20—C27—F19	144 (3)
F9—F10—F12—F11	−169 (4)	F21—F20—C27—C28	−104.0 (15)
F9—F10—F12—C14	−25.6 (10)	F21—C27—C28—O8	48.7 (10)
F9—F10—C14—F4	142.2 (9)	F21—C27—C28—C29	−129.5 (9)
F9—F10—C14—F5	118.3 (11)	O2—C2—C3—C4	1.9 (10)
F9—F10—C14—F6	69.3 (14)	O3—C8—C9—C10	177.7 (5)
F9—F10—C14—F7	27.2 (10)	O3—C11—C12—C13	64.6 (7)
F9—F10—C14—F8	6.9 (9)	O4—C17—C18—C19	−167.8 (5)
F9—F10—C14—F11	171 (2)	O4—C17—C18—C23	10.8 (7)
F9—F10—C14—F12	153.2 (11)	O5—C15—C16—C17	−0.1 (10)
F9—F10—C14—C15	−100.7 (8)	O6—C21—C22—C23	178.6 (5)
F9—F11—F12—F4	−21 (8)	O6—C24—C25—C26	177.6 (5)
F9—F11—F12—F5	−5 (3)	O7—C30—C31—C32	174.3 (5)
F9—F11—F12—F10	7 (2)	O7—C30—C31—C36	−5.7 (7)
F9—F11—F12—C14	−26.4 (10)	O8—C28—C29—C30	−2.1 (10)
F9—F11—C14—F4	143.5 (7)	O9—C34—C35—C36	179.0 (5)
F9—F11—C14—F5	128.7 (9)	O9—C37—C38—C39	−178.0 (6)
F9—F11—C14—F6	96.5 (13)	O11—C4—C5—C6	164.2 (5)
F9—F11—C14—F7	37.2 (11)	O11—C4—C5—C10	−15.1 (7)
F9—F11—C14—F8	9.2 (8)	C1—C2—C3—C4	−175.3 (5)
F9—F11—C14—F10	−7.4 (19)	C2—C3—C4—O11	−5.2 (9)
F9—F11—C14—F12	144.6 (15)	C2—C3—C4—C5	174.1 (5)
F9—F11—C14—C15	−108.6 (7)	C3—C4—C5—C6	−15.2 (8)
F9—C14—C15—O5	−46.6 (9)	C3—C4—C5—C10	165.5 (5)
F9—C14—C15—C16	132.5 (8)	C4—C5—C6—C7	179.6 (5)
F10—F9—F11—F4	−169 (4)	C4—C5—C10—C9	−179.1 (5)
F10—F9—F11—F12	−166 (5)	C5—C6—C7—C8	−0.8 (8)
F10—F9—F11—C14	163 (4)	C6—C5—C10—C9	1.6 (8)
F10—F9—C14—F4	−61.8 (15)	C6—C7—C8—O3	−177.3 (5)
F10—F9—C14—F5	−105.6 (13)	C6—C7—C8—C9	2.4 (8)
F10—F9—C14—F6	−137.2 (9)	C7—C8—C9—C10	−2.0 (8)
F10—F9—C14—F7	−149.3 (11)	C8—O3—C11—C12	−169.9 (5)
F10—F9—C14—F8	−166.2 (18)	C8—C9—C10—C5	0.0 (8)
F10—F9—C14—F11	−3.9 (10)	C10—C5—C6—C7	−1.1 (8)
F10—F9—C14—F12	−23.0 (10)	C11—O3—C8—C7	171.3 (5)
F10—F9—C14—C15	102.2 (8)	C11—O3—C8—C9	−8.3 (8)
F10—F11—F12—F4	−28 (9)	C14—F4—F5—F6	18 (3)
F10—F11—F12—F5	−12 (5)	C14—F4—F5—F7	22.0 (10)
F10—F11—F12—C14	−34 (3)	C14—F4—F5—F12	26.9 (16)
F10—F11—C14—F4	151 (2)	C14—F4—F6—F5	−159 (4)
F10—F11—C14—F5	136 (2)	C14—F4—F6—F7	26.9 (9)
F10—F11—C14—F6	104 (2)	C14—F4—F6—F8	23.3 (6)
F10—F11—C14—F7	45 (2)	C14—F4—F11—F9	−26.6 (6)
F10—F11—C14—F8	17 (2)	C14—F4—F11—F10	−32 (2)
F10—F11—C14—F9	7.4 (19)	C14—F4—F11—F12	174 (8)
F10—F11—C14—F12	152 (3)	C14—F4—F12—F5	−29.8 (18)
F10—F11—C14—C15	−101.2 (19)	C14—F4—F12—F10	−23.7 (17)
F10—F12—C14—F4	164.5 (12)	C14—F4—F12—F11	−6 (7)
F10—F12—C14—F5	150.1 (9)	C14—F5—F6—F4	19 (4)
F10—F12—C14—F6	131.7 (10)	C14—F5—F6—F7	25.0 (14)
F10—F12—C14—F7	84.9 (15)	C14—F5—F6—F8	20.9 (9)
F10—F12—C14—F8	45.0 (14)	C14—F5—F7—F6	−146.2 (19)
F10—F12—C14—F9	22.3 (10)	C14—F5—F7—F8	21 (2)
F10—F12—C14—F11	−14.0 (14)	C14—F5—F7—F9	30.7 (8)
F10—F12—C14—C15	−100.5 (9)	C14—F5—F12—F4	144 (2)
F10—C14—C15—O5	11.7 (10)	C14—F5—F12—F10	−31.4 (9)
F10—C14—C15—C16	−169.3 (8)	C14—F5—F12—F11	−26 (2)
F11—F4—F5—F6	−6 (4)	C14—F6—F7—F5	26.7 (15)
F11—F4—F5—F7	−1.9 (19)	C14—F6—F7—F8	1 (6)
F11—F4—F5—F12	3.0 (10)	C14—F6—F7—F9	22.4 (14)
F11—F4—F5—C14	−23.9 (11)	C14—F6—F8—F7	−179 (6)
F11—F4—F6—F5	175 (4)	C14—F6—F8—F9	33.0 (17)
F11—F4—F6—F7	0.6 (12)	C14—F7—F8—F6	1 (6)
F11—F4—F6—F8	−3.0 (8)	C14—F7—F8—F9	38 (3)
F11—F4—F6—C14	−26.3 (6)	C14—F7—F9—F8	−137 (3)
F11—F4—F12—F5	−24 (8)	C14—F7—F9—F10	30.1 (10)
F11—F4—F12—F10	−18 (6)	C14—F7—F9—F11	27.3 (7)
F11—F4—F12—C14	6 (7)	C14—F8—F9—F7	36 (3)
F11—F4—C14—F5	152.5 (14)	C14—F8—F9—F10	19 (2)
F11—F4—C14—F6	146.4 (8)	C14—F8—F9—F11	17.1 (14)
F11—F4—C14—F7	124.4 (9)	C14—F9—F10—F11	15 (4)
F11—F4—C14—F8	102.7 (13)	C14—F9—F10—F12	23.8 (9)
F11—F4—C14—F9	58.5 (13)	C14—F9—F11—F4	27.5 (6)
F11—F4—C14—F10	12.2 (8)	C14—F9—F11—F10	−163 (4)
F11—F4—C14—F12	−0.9 (11)	C14—F9—F11—F12	30.6 (13)
F11—F4—C14—C15	−105.6 (7)	C14—F10—F11—F4	25.2 (15)
F11—F9—F10—F12	9 (3)	C14—F10—F11—F9	13 (3)
F11—F9—F10—C14	−15 (4)	C14—F10—F11—F12	30 (3)
F11—F9—C14—F4	−57.8 (13)	C14—F10—F12—F4	25.0 (18)
F11—F9—C14—F5	−101.7 (12)	C14—F10—F12—F5	27.9 (8)
F11—F9—C14—F6	−133.2 (8)	C14—F10—F12—F11	−143 (3)
F11—F9—C14—F7	−145.3 (9)	C14—F11—F12—F4	6 (7)
F11—F9—C14—F8	−162.3 (16)	C14—F11—F12—F5	21.2 (19)
F11—F9—C14—F10	3.9 (10)	C14—F11—F12—F10	34 (3)
F11—F9—C14—F12	−19.1 (8)	C14—C15—C16—C17	−179.0 (5)
F11—F9—C14—C15	106.2 (8)	C15—C16—C17—O4	4.8 (9)
F11—F10—F12—F4	168 (4)	C15—C16—C17—C18	−177.1 (5)
F11—F10—F12—F5	171 (3)	C16—C17—C18—C19	13.9 (8)
F11—F10—F12—C14	143 (3)	C16—C17—C18—C23	−167.5 (5)
F11—F10—C14—F4	−29 (2)	C17—C18—C19—C20	179.3 (5)
F11—F10—C14—F5	−53 (2)	C17—C18—C23—C22	−179.6 (5)
F11—F10—C14—F6	−102 (2)	C18—C19—C20—C21	−0.3 (8)
F11—F10—C14—F7	−144 (2)	C19—C18—C23—C22	−0.9 (8)
F11—F10—C14—F8	−164 (2)	C19—C20—C21—O6	−178.9 (5)
F11—F10—C14—F9	−171 (2)	C19—C20—C21—C22	0.0 (8)
F11—F10—C14—F12	−17.8 (18)	C20—C21—C22—C23	−0.1 (8)
F11—F10—C14—C15	88 (2)	C21—O6—C24—C25	175.4 (5)
F11—F12—C14—F4	178.5 (19)	C21—C22—C23—C18	0.6 (9)
F11—F12—C14—F5	164.1 (15)	C23—C18—C19—C20	0.7 (8)
F11—F12—C14—F6	145.7 (14)	C24—O6—C21—C20	−172.6 (5)
F11—F12—C14—F7	98.9 (18)	C24—O6—C21—C22	8.6 (8)
F11—F12—C14—F8	59.0 (18)	C27—F13—F14—F21	10 (6)
F11—F12—C14—F9	36.3 (15)	C27—F13—F15—F16	−32.4 (10)
F11—F12—C14—F10	14.0 (14)	C27—F13—F21—F14	−11 (7)
F11—F12—C14—C15	−86.5 (15)	C27—F13—F21—F19	33.1 (11)
F11—C14—C15—O5	38.6 (9)	C27—F13—F21—F20	27 (3)
F11—C14—C15—C16	−142.3 (8)	C27—F14—F21—F13	168 (7)
F12—F4—F5—F6	−9 (5)	C27—F14—F21—F19	30.0 (10)
F12—F4—F5—F7	−5 (2)	C27—F14—F21—F20	21 (2)
F12—F4—F5—C14	−26.9 (16)	C27—F15—F16—F18	−29.7 (11)
F12—F4—F6—F5	172 (4)	C27—F16—F18—F17	171 (15)
F12—F4—F6—F7	−1.9 (15)	C27—F16—F18—F19	−15.7 (19)
F12—F4—F6—F8	−5.5 (12)	C27—F16—F18—F20	−15.1 (12)
F12—F4—F6—C14	−28.7 (9)	C27—F17—F18—F16	−9 (15)
F12—F4—F11—F9	160 (8)	C27—F17—F18—F19	−17 (4)
F12—F4—F11—F10	154 (9)	C27—F17—F18—F20	−16 (3)
F12—F4—F11—C14	−174 (8)	C27—F17—F19—F18	158 (6)
F12—F4—C14—F5	153.3 (18)	C27—F17—F19—F20	−18 (4)
F12—F4—C14—F6	147.2 (11)	C27—F17—F19—F21	−32.4 (11)
F12—F4—C14—F7	125.3 (12)	C27—F18—F19—F17	−19 (5)
F12—F4—C14—F8	103.6 (15)	C27—F18—F19—F20	−14 (6)
F12—F4—C14—F9	59.3 (16)	C27—F18—F19—F21	−30.8 (13)
F12—F4—C14—F10	13.1 (11)	C27—F18—F20—F19	164 (6)
F12—F4—C14—F11	0.9 (11)	C27—F18—F20—F21	−38.8 (16)
F12—F4—C14—C15	−104.7 (10)	C27—F19—F20—F18	−12 (5)
F12—F5—F6—F4	−5 (3)	C27—F19—F20—F21	−38 (3)
F12—F5—F6—F7	1 (2)	C27—F19—F21—F13	−33.1 (11)
F12—F5—F6—F8	−3.0 (15)	C27—F19—F21—F14	−26.7 (9)
F12—F5—F6—C14	−23.9 (9)	C27—F19—F21—F20	138 (3)
F12—F5—F7—F6	−179.0 (18)	C27—F20—F21—F13	−23 (2)
F12—F5—F7—F8	−11 (2)	C27—F20—F21—F14	−15.8 (17)
F12—F5—F7—F9	−2.1 (9)	C27—F20—F21—F19	−36 (3)
F12—F5—F7—C14	−32.8 (6)	C27—C28—C29—C30	175.8 (5)
F12—F5—C14—F4	−19.0 (13)	C28—C29—C30—O7	3.2 (8)
F12—F5—C14—F6	151.8 (12)	C28—C29—C30—C31	−175.1 (5)
F12—F5—C14—F7	134.5 (9)	C29—C30—C31—C32	−7.3 (8)
F12—F5—C14—F8	126.0 (10)	C29—C30—C31—C36	172.7 (5)
F12—F5—C14—F9	97.9 (13)	C30—C31—C32—C33	179.6 (5)
F12—F5—C14—F10	29.7 (10)	C30—C31—C36—C35	−178.7 (5)
F12—F5—C14—F11	8.9 (9)	C31—C32—C33—C34	−0.6 (8)
F12—F5—C14—C15	−110.6 (8)	C32—C31—C36—C35	1.3 (8)
F12—F10—F11—F4	−4.6 (16)	C32—C33—C34—O9	−178.0 (5)
F12—F10—F11—F9	−17 (6)	C32—C33—C34—C35	0.7 (9)
F12—F10—F11—C14	−30 (3)	C33—C34—C35—C36	0.2 (9)
F12—F10—C14—F4	−11.0 (9)	C34—O9—C37—C38	−175.1 (5)
F12—F10—C14—F5	−34.9 (12)	C34—C35—C36—C31	−1.2 (9)
F12—F10—C14—F6	−83.9 (15)	C36—C31—C32—C33	−0.4 (8)
F12—F10—C14—F7	−126.0 (10)	C37—O9—C34—C33	−7.1 (9)
F12—F10—C14—F8	−146.3 (10)	C37—O9—C34—C35	174.1 (5)
F12—F10—C14—F9	−153.2 (11)		

### Aqua(ethanol-κO)tris[4,4,4-trifluoro-1-(4-propoxyphenyl)butane-1,3-dionato(1-)-κ2O,O']europium(III) Hydrogen-bond geometry (Å, º)

**Table:**

*D*—H···*A*	*D*—H	H···*A*	*D*···*A*	*D*—H···*A*
O1—H1···O5	0.84	2.47	2.761 (5)	101
O1—H1···O6^i^	0.84	2.17	2.978 (6)	161
O10—H10*B*···O9^ii^	0.85	2.15	2.866 (6)	142

Symmetry codes: (i) −*x*+1, −*y*+1, −*z*; (ii) −*x*+1/2, *y*−1/2, −*z*+1/2.
